# Rare variants in optic disc area gene *CARD10* enriched in primary open‐angle glaucoma

**DOI:** 10.1002/mgg3.248

**Published:** 2016-10-03

**Authors:** Tiger Zhou, Emmanuelle Souzeau, Shiwani Sharma, Owen M. Siggs, Ivan Goldberg, Paul R. Healey, Stuart Graham, Alex W. Hewitt, David A. Mackey, Robert J. Casson, John Landers, Richard Mills, Jonathan Ellis, Paul Leo, Matthew A. Brown, Stuart MacGregor, Kathryn P. Burdon, Jamie E. Craig

**Affiliations:** ^1^ Department of OphthalmologyFlinders UniversityAdelaideSAAustralia; ^2^ Discipline of OphthalmologyUniversity of SydneySydneyNSWAustralia; ^3^ Glaucoma UnitSydney Eye HospitalSydneyNSWAustralia; ^4^ Menzies Institute for Medical ResearchUniversity of TasmaniaHobartTASAustralia; ^5^ Centre for Ophthalmology and Visual ScienceLions Eye InstituteUniversity of Western AustraliaPerthWAAustralia; ^6^ Discipline of Ophthalmology & Visual SciencesUniversity of AdelaideAdelaideSAAustralia; ^7^ Diamantina InstituteTranslational Research InstitutePrincess Alexandra HospitalUniversity of QueenslandWoolloongabbaQLDAustralia; ^8^ Statistical GeneticsQIMR Berghofer Medical Research InstituteRoyal Brisbane HospitalBrisbaneQLDAustralia

**Keywords:** *CARD10*, genome‐wide association study, rare variants, whole exome sequencing

## Abstract

**Background:**

Genome‐wide association studies (GWAS) have identified association of common alleles with primary open‐angle glaucoma (POAG) and its quantitative endophenotypes near numerous genes. This study aims to determine whether rare pathogenic variants in these disease‐associated genes contribute to POAG.

**Methods:**

Participants fulfilled strict inclusion criteria of advanced POAG at a young age of diagnosis. Myocilin mutation carriers were excluded using direct sequencing. Whole exome sequencing was performed on 187 glaucoma cases and 103 local screened nonglaucoma controls then joint‐called with exomes of 993 previously sequenced Australian controls. GWAS‐associated genes were assessed for enrichment of rare predicted pathogenic variants in POAG. Significantly enriched genes were compared against Exome Aggregation Consortium (ExAC) public control.

**Results:**

Eighty‐six GWAS disease or trait‐associated glaucoma genes were captured and sequenced. *CARD10* showed enrichment after Bonferroni correction for rare variants in glaucoma cases (OR = 13.2, *P* = 6.94 × 10^−5^) with mutations identified in 4.28% of our POAG cohort compared to 0.27% in controls. *CARD10* was significantly associated with optic disc parameters in previous GWAS. The whole GWAS gene set showed no enrichment in POAG overall (OR = 1.12, *P* = 0.51).

**Conclusion:**

We report here an enrichment of rare predicted pathogenic coding variants within a GWAS‐associated locus in POAG (*CARD10*). These findings indicate that both common and rare pathogenic coding variants in *CARD10* may contribute to POAG pathogenesis.

## Introduction

Globally, glaucoma is the most common cause of irreversible and preventable blindness and the second most common cause of all blindness after cataract, accounting for 8% of blindness worldwide in 2010 (Pascolini and Mariotti 2012). The defining feature of glaucoma is an optic neuropathy characterized by optic disc cupping with corresponding visual field loss (Foster et al. 2002). With early identification and intervention, mainly with intraocular pressure (IOP)‐lowering therapy, vision loss is usually preventable (Leske et al. 2003). The disease incorporates a spectrum of subtypes ranging from congenital to age‐related, normal of raised IOP, open or closed iridocorneal angle and primary or secondary etiology (Kwon et al. 2009). Primary open‐angle glaucoma (POAG) is the most common disease subset and the high IOP phenotype is predominant in Caucasian and African ethnicities (Tham et al. 2014). While elevated IOP is the single greatest risk factor for POAG, other associated endophenotypes include optic disc morphology and central corneal thickness (CCT) (Miglior et al. 2007; Jiang et al. 2012; Hollands et al. 2013).

POAG exhibits high heritability, with family history conferring up to a tenfold increase in disease risk, implying a genetic etiology (Wolfs et al. 1998). Several genes with high penetrance mutations have been discovered through family‐based linkage studies (*MYOC* ‐ *601652, *OPTN* ‐ *602432, *TBK1* ‐ *604834, *CYP1B1* ‐ *601771), although collectively they account for <5% of total disease burden in POAG (Fingert 2011). The common disease, common variant hypothesis has been tested in POAG. Genome‐wide association studies (GWAS) suggest at least part of the disease burden is attributable to common variants near or within a number of genes including *ABCA1* (+600046) (Chen et al. 2014; Gharahkhani et al. 2014; Hysi et al. 2014), *AFAP1* (*608252) (Gharahkhani et al. 2014), *ATOH7* (*609875) (Ramdas et al. 2011), *CAV1*‐*CAV2* (*601047; *601048) (Thorleifsson et al. 2010; Wiggs et al. 2011; Hysi et al. 2014), *CDKN2B‐AS1* (*613149) (Burdon et al. 2011; Nakano et al. 2012; Osman et al. 2012), *GAS7* (*603127) (Hysi et al. 2014), *GMDS* (*602884) (Gharahkhani et al. 2014), *PMM2* (*601785) (Chen et al. 2014), *SIX1‐SIX6* (*601205; *606326) (Ramdas et al. 2011; Osman et al. 2012; Wiggs et al. 2012), and *TMCO1* (*614123) (Burdon et al. 2011; van Koolwijk et al. 2012; Hysi et al. 2014). Some POAG risk loci have shown significant associations with quantitative traits related to the disease such as IOP, optic disc morphology, and central corneal thickness (Lu et al. 2013; Hysi et al. 2014; Springelkamp et al. 2014). However the magnitude of odds ratios for all genome‐wide associated disease polymorphisms are less than two, even for the strongest loci including *TMCO1* (Burdon et al. 2011) and *CDKN2B‐AS1* (Burdon et al. 2011; Wiggs et al. 2012), with a significant proportion of normal controls also carrying the risk alleles at associated polymorphisms (Mackey and Hewitt 2014). Furthermore, the majority of the GWAS signals are located in the noncoding regions of the genome. There is as yet no clear understanding of the mechanism of association of these noncoding polymorphisms with disease etiology (Manolio et al. 2009).

The exact contribution of rare variants to glaucoma is not known. Common tagged single‐nucleotide polymorphism (SNP) analysis at the genome level, using SNP microarray technology cannot ascertain the burden of rare variants due to the poor linkage disequilibrium between common and rare variants (Siu et al. 2011). This case–control study utilized whole exome sequencing (WES) to investigate the degree of enrichment and proportion of disease burden accounted for by rare pathogenic variants in genes near common variants known to be associated with POAG and related quantitative traits from GWAS as this has not been adequately addressed in the literature.

## Materials and Methods

This study adhered to the principles listed by the revised Declaration of Helsinki and the Australian National Health and Medical Research Council (NHMRC) statement of ethical conduct in research involving humans. Ethical approval was obtained from the Southern Adelaide and Flinders University Clinical Research Ethics Committee. All peripheral blood samples were collected for genomic DNA extraction as a part of the Australian and New Zealand Registry of Advanced Glaucoma (ANZRAG) as previously described (Souzeau et al. 2012).

### Participants

Unrelated Caucasian participants with advanced glaucoma and nonglaucoma Caucasian controls were included for the study. All DNA samples were extracted from peripheral blood samples, using the QIAamp^®^ DNA blood kit (Qiagen, Hilden, Germany) following the manufacturer's protocol. Our case cohort inclusion criteria selected only participants with the most severe disease and the youngest age (mean = 44.4 years, SD = 10.4 years) of onset within the ANZRAG database. For this study, participants were included if there was glaucomatous visual field loss involving at least two of the four central fixation squares with a pattern standard deviation of <0.5% on a reliable Humphrey 24‐2 field (Carl Zeiss, Dublin, CA), or a mean deviation of worse than −15 dB in the worst affected eye. For participants without formal visual field testing, their best‐corrected visual acuity had to be worse than 20/200 due to glaucomatous damage. Furthermore glaucomatous damage had to also be present in the less affected eye as measured by a reliable Humphrey 24‐2 field, with corresponding neuroretinal rim thinning. With the ANZRAG database participants with the youngest age of diagnosis were selected for this study. Efforts were made to ensure a comparable split of participants with high and normal IOP. High‐tension glaucoma (HTG) was defined as having a maximum recorded IOP of >21 mmHg. Individuals with known *MYOC* mutations were excluded from this study as the genetic cause for their POAG has already been identified.

For the local control cohort, all participants were examined to exclude glaucoma or glaucoma‐related phenotypes. Furthermore, in silico analysis included a larger unexamined control cohort from the Australian Osteoporosis Genetics Consortium (Estrada et al. 2012) (AOGC). These controls were female participants with high or low bone mass who were otherwise healthy by self‐report.

### Data acquisition

Exon capture and enrichment was performed on all glaucoma cases and local controls with the SureSelect Human All Exon V4 enrichment kit (Agilent, Santa Clara, CA, USA) as per manufacturer's protocol and enriched DNA sequenced on a HiSeq2000 (Illumina, San Diego, CA, USA) with paired end 100 bp reads (Macrogen Next Generation Sequencing Services). Local controls served as both technical and phenotypic controls. Raw experimental data were cleaned and joint‐called with AOGC exome data that were captured with Nimblegen Human Exome Capture V2 (Roche, Basel, Switzerland) and sequenced on the HiSeq2000 (Illumina, San Diego, CA, USA) (University of Queensland Centre for Clinical Genomics). FASTQ files were aligned to human genome build hg19 with novoalign (version 3.02.08). Duplicate reads were marked with Picard MarkDuplicates (version 1.124). Local indel realignment and base quality recalibration were performed with GATK (McKenna et al. 2010) (version 3.2‐2). Variant calling of single‐nucleotide variants and small indels was performed with the UnifiedGenotyper module in GATK and variant quality scores were recalibrated according to the GATK “Best Practices Guidelines” (DePristo et al. 2011; Van der Auwera et al. 2013). Annotation of calls was performed using ANNOVAR (Wang et al. 2010) software using refGene, SIFT, PolyPhen2 HVAR, Exome Sequencing Project (ESP), 1000 Genomes and Exome Aggregation Consortium (ExAC) databases.

### Data analysis

Using in‐house UNIX scripts, only protein coding exonic and splicing site variants were selected for analysis. Genes were included in the list if they were reported by any published GWAS or meta‐analysis paper to be the closest to SNPs associated with POAG or disease endophenotype at *P* < 5 × 10^−8^ in any ethnicity. Quality control threshold was set at a Genotype Quality (GQ) score of 20. Focusing on the impact of rare mutations, all variants with known minor allele frequency (MAF) of >1% in dbSNP, 1000 genomes and ExAC were excluded. Pathogenicity filtering removed all synonymous variants and missense variants considered tolerated or only possibly damaging by both SIFT and PolyPhen‐2 HVAR, and retained stop site, frameshift and predicted deleterious missense mutations. Raw VCF data from the Exome Aggregation Consortium were annotated with the ANNOVAR pipeline followed by variant selection using the same in‐house scripts to target only rare deleterious mutations in the non‐Finnish European subgroup to act as a larger in silico population control. Mutation loads per gene were calculated for the glaucoma cases, local control, AOGC control and ExAC control cohorts by summing the minor allele counts of all qualifying variants in the same gene and dividing by the average number of captured alleles for those variants, thereby adjusting for capture rate. Odds ratios were generated between cases and each control cohort separately, and Fisher's exact test used to calculate *P*‐values, and Bonferroni correction applied for multiple testing of all analyzed genes (*n* = 86, threshold *P* = 5.81 × 10^−4^). Five sets of randomly chosen independent groups of 86 WES‐captured genes were selected using a random number generator and analyzed using the same protocol to act as control gene sets. This step further strengthens the positive results by examining the likelihood of false‐positive findings.

### Validation of variants

All variants within significantly associated genes were independently validated by capillary sequencing (primers listed in Table S1). PCRs were conducted on carrier samples using 40 ng of DNA, 10 μmol of each corresponding forward and reverse primers, 0.5 units of HotStarTaq DNA polymerase (Qiagen, Hilden, Germany), 2 μmol of dNTP and PCR buffer in a 20 μL volume per reaction. Thirty cycles were performed at an annealing temperature of 62°C. Five μL of each PCR product was incubated with 2 μL of Shrimp Alkaline Phosphatase (Affymetrix, Santa Clara, CA, USA) and 0.5 μL of *E. coli* exonuclease I (New England BioLabs, Ipswich, MA, USA) at 37°C for 60 min. Sequencing was carried out on the fluorescence‐based capillary electrophoresis system 3130xl Genetic Analyzer (Life Technologies, Carlsbad, CA, USA) using BigDye Terminator V3.1 (Life Technologies, Carlsbad, CA, USA) according to manufacturer's protocol.

## Results

A total of 187 unrelated participants with advanced POAG and 103 screened nonglaucoma controls were sequenced with whole exome capture and enrichment. Combined with the large unscreened cohort of AOGC controls (*n* = 993), the total number of controls was 1096. More participants had high‐tension glaucoma (HTG, *n* = 122) than normal‐tension glaucoma (NTG, *n* = 65). To date, 101 genes have been reported by GWAS studies as being near SNPs that are statistically associated with POAG, central corneal thickness, IOP or optic disc morphology. Of these 101 genes, 86 had qualifying (MAF <0.01 and predicted pathogenic) variants captured in glaucoma and control cohorts in this study (Table S2).

The sequencing strategy utilized for cases and local controls in this study yielded good read quality, coverage, and depth. Mean percentage of mappable reads was high at 99.4% with an average total on‐target reads per sample of 4.12 × 10^9^ and an average depth of 73 reads per target base. Average coverage at >10× depth was 97.9% of all targeted exonic regions. The average numbers of coding SNPs and indels per participant was 19,605 and 465, respectively. AOGC controls had an average depth of 24 reads per target base and 10× coverage of 75.1%. After filtering for rare predicted pathogenic variants only, there was an average of 159 qualifying variants per participant. A total of 1159 variants were within the 86 included genes in case, local or AOGC control cohorts. There was no significant enrichment (OR = 1.12, *P* = 0.51) in the total carrier rate of qualifying variants in this gene set in glaucoma cases (64.2%, 120 individuals) compared with local and AOGC controls (61.5%, 648 individuals) although there was a trend towards increased mutation load in POAG cases (Table S3).

Four genes (*CARD10*,* CWC27*,* RERE*, and *USP37*) were nominally enriched (uncorrected *P* < 0.05) in the glaucoma cohort (Table 1). Only *CARD10* (caspase recruitment domain containing protein 10) remained statistically significant after Bonferroni correction for 86 tested genes (*P* = 6.94 × 10^−5^, corrected *P* = 5.97 × 10^−3^) with an odds ratio of 13.2 (3.5–50.2). Eight out of 187 POAG cases (4.28%) carried a rare predicted pathogenic variant in *CARD10* confirmed by Sanger sequencing (Figure S1). Comparatively, qualifying variants in *CARD10* were carried by 0.27% (3/1096) of the joint‐called controls. A total of five nonsynonymous variants in this gene were identified in the POAG cohort with all but one absent in the controls (Table 2). These variants were predicted to be pathogenic by SIFT or PolyPhen2 as per the filtering criteria. Additionally, the variants were located in highly conserved regions, all having GERP (Genomic Evolutionary Rate Profiling) scores of >2 (mean GERP score = 4.8). When tested against qualifying variants in the unscreened public domain ExAC non‐Finnish European cohort (*n* = 33370) filtered with our pipeline, *CARD10* remained significantly enriched in the glaucoma cohort, albeit with a lower odds ratio (OR = 3.3, *P* = 3.9 × 10^−3^). *CARD10* variant carriers had younger age at diagnosis (34.9 vs. 44.8 years, *P* = 0.36) and higher IOP (27.4 vs. 24.8, *P* = 0.11) than non‐*CARD10* cases, although the difference was not statistically significant due to the small number of *CARD10* carriers. All eight *CARD10* qualifying variants were successfully validated in the carrier cases using capillary sequencing (Figure S1) and submitted to ClinVar database (http://www.ncbi.nlm.nih.gov/clinvar/) with accessions SCV000266585–SCV000266589. Subanalyses of the HTG and NTG cohorts with local and AOGC controls showed significant association of *CARD10* variants with HTG subgroup (OR = 15.2, corrected *P* = 0.01). Five control gene sets of 86 randomly selected genes were examined and none contained any gene that was significantly associated with POAG after Bonferroni correction (Tables S4–S8).

**Table 1:** Genes at genome‐wide significant associated loci that reached nominal significance for enrichment of rare variants in the glaucoma cohort. Mutational load represents the total allele frequency burden of qualifying variants in each gene. Odds ratios are adjusted for capture quality described in the Methods section. *P*‐values are corrected using Bonferroni multiple testing correction

Gene	POAG load (%)	Control load (%)	Odds ratio	*P*‐value	Corrected *P*‐value
*CARD10*	2.14	0.14	13.19	6.94 × 10^−5^	5.97 × 10^−3^
*CWC27*	1.34	0.14	6.89	9.47 × 10^−3^	0.81
*RERE*	1.60	0.23	5.49	0.0114	0.98
*USP37*	1.87	0.18	7.32	5.11 × 10^−3^	0.44

**Table 2:** Qualifying *CARD10* (NM_014550.3) variants captured in glaucoma cohort and controls. SIFT score <0.05 is predicted damaging. PolyPhen2 HVAR score >0.909 is predicted damaging

cDNA	Residue	SIFT	PolyPhen2	POAG cases	POAG freq	Control freq	ExAC freq
c.635G>A	p.Arg212His	0.10	0.987	1	2.7 × 10^−3^	0	0
c.983C>T	p.Ala328Val	0.02	0.944	3	8.0 × 10^−3^	9.1 × 10^−4^	3.6 × 10^−3^
c.1024G>A	p.Val342Met	0.04	0.025	1	2.7 × 10^−3^	0	2 × 10^−4^
c.1210C>T	p.Arg404Trp	0.01	0.764	1	2.7 × 10^−3^	0	1.5 × 10^−5^
c.2485C>T	p.Arg829Trp	0.02	0.01	2	5.3 × 10^−3^	0	4.0 × 10^−4^
c.3081delC	p.Pro1027fs			0	0	4.6 × 10^−4^	0

## Discussion

Genome‐wide association studies have been examined for common variants linked to POAG, NTG and various associated endophenotypes; IOP, vertical cup‐to‐disc ratio (VCDR), neuroretinal rim area, optic disc area and central corneal thickness. Poor linkage disequilibrium exists between common and rare variants; this has been demonstrated experimentally (Siu et al. 2011). For this reason, previous GWAS designs have reduced power to detect signals at single‐nucleotide variants (SNVs) with MAF of <1%. This study employed WES to patch the “gap” left by GWAS and to explore whether rare variants (MAF <1%) within 86 putative POAG risk loci were also enriched within a severe POAG cohort. Although the majority of rare variants from the 86 POAG risk loci do not show significant enrichment in cases, the plausibility that GWAS candidates may contribute to disease via a common disease rare variant hypothesis has been demonstrated by our study.

A previous candidate gene study of *SIX6* reported an enrichment of rare variants in POAG in that gene (Carnes et al. 2014). The most prevalent variant, with a carrier frequency of 1.6% in cases, identified by Carnes et al. (2014) (rs146737847:G>A) was found in 1.6% of our POAG cases, but also in 1.9% of local screened controls and 1.6% of AOGC controls with an overall OR of 0.98. The other three rare variants found in that study at a carrier frequency of 0.4% were not detected in our cases or controls. In summary, our study found no enrichment of rare variants in *SIX6* in our POAG cases versus controls.

### Genome‐wide association of *CARD10*

**Figure 1:**
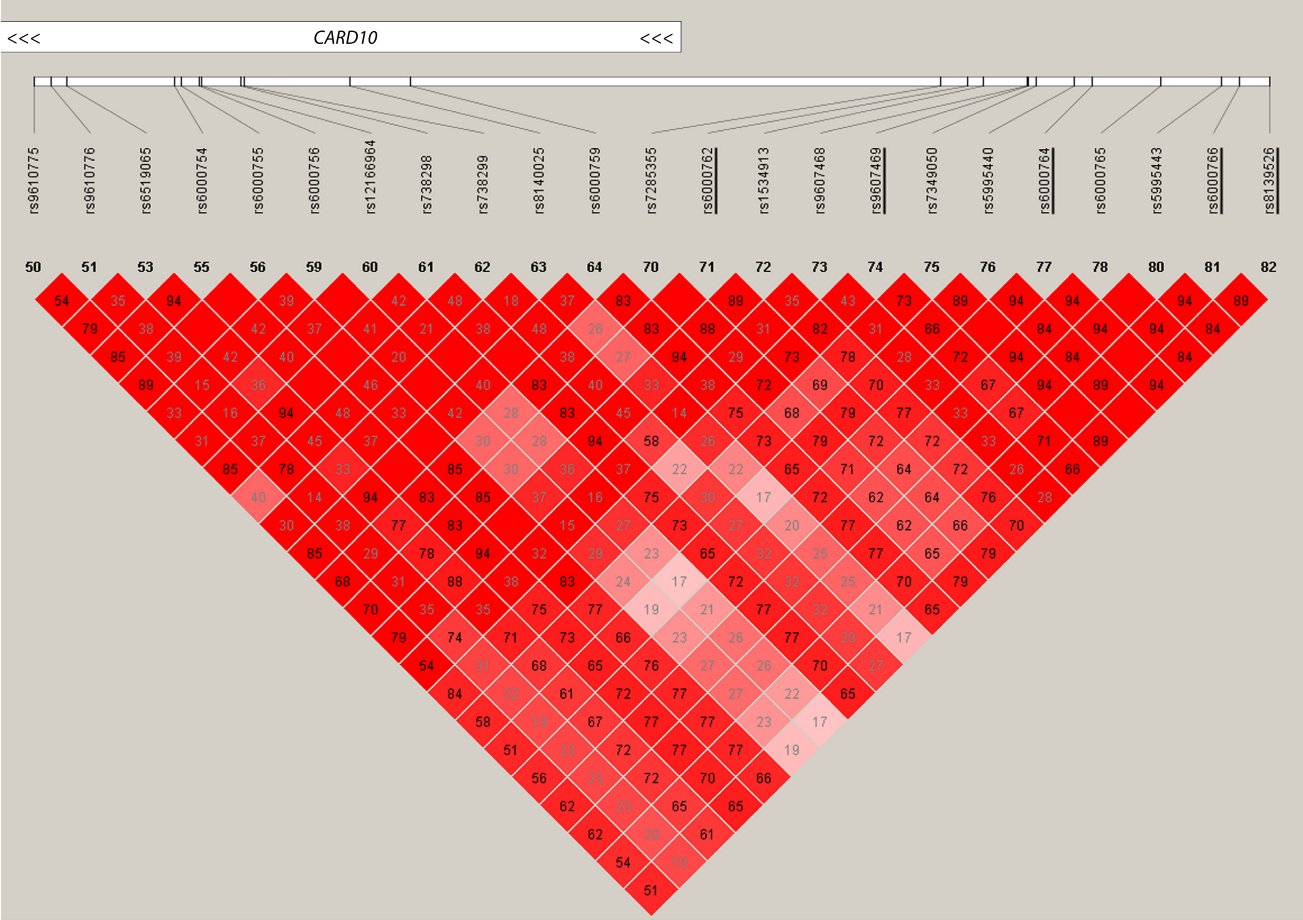
Linkage disequilibrium between GWAS SNPs and common SNPs in *CARD10* from HapMap CEU data showing D’ values. GWAS SNPs are underlined. *CARD10* gene location is represented by rectangle at the top. The left‐most SNP rs9610775 (p.Arg289Gln) represents the boundary of overlap between the GWAS significant LD block and the *CARD10* gene.

SNPs near *CARD10* were first found to be significantly associated with optic disc area on GWAS in Singaporean Asians, a result replicated in a Dutch Caucasian cohort (Khor et al. 2011). Optic disc area is relevant in POAG as a large optic disc is correlated with increased susceptibility to glaucoma in Caucasian populations (Healey and Mitchell 1999). Meta‐analysis of optic disc morphology further implicated common variants in *CARD10* in VCDR but not POAG after adjustment for optic disc area (Springelkamp et al. 2014). This result has since been replicated in a separate case–control study in an Indian population (Philomenadin et al. 2015). Cup‐to‐disc ratio is fundamental in POAG as it is often used as a diagnostic criterion due to its strong positive correlation with disease incidence (Miglior et al. 2007; Hollands et al. 2013). The SNPs from the original GWAS linking *CARD10* to optic disc area were situated between 3262 bp and 7204 bp upstream of *CARD10* (Khor et al. 2011). These SNPs are in strong linkage disequilibrium with all common SNPs up to and including rs9610775, which is a missense coding variant, p.R289Q in the *CARD10* gene with a population MAF of 13% (Fig. 1). The signal identified in the optic disc area GWAS may possibly relate to *CARD10* coding mutations. The SNPs found to be significantly associated with VCDR on meta‐analysis were 260 kb upstream of *CARD10* with no linkage disequilibrium to the gene (Springelkamp et al. 2014). More likely, these variants are involved in gene expression and regulation. Therefore, the published GWAS data suggests that both coding variants in *CARD10* and its expression level may contribute to optic disc pathology.

GWAS was able to highlight the association between regulatory and common coding SNPs in *CARD10* with crucial optic disc parameters for the development of POAG (Khor et al. 2011; Springelkamp et al. 2014; Philomenadin et al. 2015). This study complements the GWAS findings by implicating rare coding *CARD10* variants that likely disrupt gene function, and are associated with increased POAG risk within our cohort of advanced glaucoma participants. Both common and rare variants concurrently contribute to disease risk via differing mechanisms affecting the same gene ‐ *CARD10*. Although more HTG participants harbored rare *CARD10* variants than NTG participants, the presence of NTG *CARD10* variant carriers suggests that IOP elevation is not required for the potential of *CARD10* to cause glaucoma. No definitive inference can be made from this discrepancy due to the small sample size of the subcohorts. A plausible hypothesis would be that the increased susceptibility to retinal ganglion cell apoptosis in the *CARD10* mutation carrying individuals is compounded by elevated IOP to cause glaucoma at an earlier age. The participant inclusion criteria of this study selected exclusively for advanced glaucoma phenotype and young age of diagnosis resulting in an enrichment of *CARD10* variant carriers that also maintained elevated IOP.

### *CARD10* function

Discovered in 2001, *CARD10* functions as a signaling protein in the regulation of the NF*κ*B (nuclear factor kappa B) pathway via activation of membrane‐bound G protein‐coupled receptors (McAllister‐Lucas et al. 2001; Wang et al. 2001). This cascade is initiated through interactions between the N‐terminal CARD domains of CARD10, BCL10 and MALT1, which forms a CBM complex (Scudiero et al. 2014). Overexpression of *CARD10* has been demonstrated in multiple human neoplasias including bladder (Man et al. 2014), breast (Zhao et al. 2013), colon (Miao et al. 2012), lung (Li et al. 2012), ovarian (Xie et al. 2014), pancreatic (Du et al. 2014) and renal cancers (Wu et al. 2013). In all cases, the overexpression of *CARD10* is associated with increased cell survival, proliferation and therefore poor prognosis of cancer. The underlying physiology appears to involve enhanced cell cycling via NF*κ*B facilitated up regulation of cyclin D1, cyclin E, Bcl‐2 and phosphorylated I*κ*B (Miao et al. 2012; Zhao et al. 2013; Xie et al. 2014). Other documented consequences of *CARD10* upregulation include leukocyte activation (Cowan et al. 2014) and inhibition of angiogenesis (Rau et al. 2014). Homozygous knockout of *CARD10* in mice causes nonviability through neural tube defects (Grabiner et al. 2007). Grabiner et al. (2007) further demonstrated that *CARD10* may be required in neural crest cell survival through G protein‐coupled receptor induction of NF*κ*B activation.

Primary open‐angle glaucoma is a disease of enhanced retinal ganglion cell apoptosis (Foster et al. 2002). The NF*κ*B pathway is profoundly involved in regulation of cellular apoptosis. Traditionally, it was only thought to be a promoter of cell survival and proliferation via downstream transcription of anti‐apoptotic proteins. As such, overexpression of NF*κ*B leads to enhanced growth of cancerous cells (Escarcega et al. 2007). However more recent evidence in the central nervous system suggests NF*κ*B may also play a proapoptotic role depending on the nature of the noxious stimuli (Kaltschmidt et al. 2005). In certain situations such as neuronal ischemia and Parkinson's disease, upregulation of NF*κ*B led to neuronal death via p53 signaling. Evidence in tumor cells indicates that *CARD10* promotes the antiapoptotic effect of NF*κ*B signaling. Therefore loss of function or downregulation of *CARD10* leads to increased apoptosis, especially in mice neural crest cells (Grabiner et al. 2007). Conversely, proapoptotic activity of NF*κ*B signalling has been recognized in the rat retina. NMDA (N‐methyl‐d‐aspartate)‐induced expression of NF*κ*B p65 led to retinal ganglion cell death which was ameliorated with knockdown of p65 by antisense oligonucleotides (Kitaoka et al. 2007). Retinal ganglion cell damage from NF*κ*B is likely due to glial cell activation and interleukin (IL)‐1*β* secretion (Kitaoka et al. 2007). Given the intimate and complex relationship between *CARD10*, NF*κ*B and apoptosis, pathogenic coding mutations in *CARD10* are likely to affect apoptosis signaling. The coding mutations identified within the glaucoma cohort in this study have not been previously characterized, however they may augment retinal ganglion cell death via up or down‐regulation of NF*κ*B. This distinction is important to ascertain in order to translate these findings toward therapeutic strategies in glaucoma management.

### Strengths and limitations

A strength of this study is the enrichment of the disease cohort with well‐selected extreme disease phenotypes, and it is the largest study to examine whole exomes of a well matched case–control cohort of this nature in the glaucoma field. ANZRAG is the largest collection advanced glaucoma patients worldwide. Our controls are hypernormal and have been individually examined to ensure the absence of glaucoma. Finally, all significantly enriched variants were confirmed via capillary sequencing thereby eliminating the possibility of false‐positive calls.

This study has some limitations which were largely technical. Whole exome sequencing has enabled the rapid examination of large numbers of genes at a reasonable cost. Its advantage over microarray technology for genetic studies lies in its ability to capture rare coding variants. However this also introduces the challenge of variable capture. AOGC samples were sequenced on a different platform with a different capture probe set to the case cohort. Joint‐calling of the two datasets was used to limit the amount of artifact generated *in silico*. However, capture‐ and sequencing platform‐specific differences remained. Some difference in capture rate at specific locations was evident and translated to incomplete capture of some variants in the AOGC cohort. Adjustment for the capture rate was performed in the calculation of odds ratios and *P*‐values. ExAC samples are more heterogeneous in data acquisition compared with this study cohort. Inability to joint‐call our data with ExAC may have overestimated the actual mutation burden in this public domain control cohort. Hence the odds ratio between case and controls falls considerably when ExAC data is included and may represent an underestimation. Alternatively, incomplete coverage of AOGC controls may have contributed to an overestimation of the discovery odds ratio. Nevertheless, a statistically significant difference remained between POAG and controls in *CARD10* mutational burden even with comparison with ExAC controls.

## Conclusion

Overall, the majority of genes near common variants associated with glaucoma and its endophenotypes found in GWAS did not show enrichment of rare variants in POAG. Rare predicted pathogenic variants in *CARD10*, a gene located near SNPs associated with optic disc area and VCDR in genome‐wide association studies, were enriched in a cohort of advanced POAG. Further research is required to elucidate the exact influence of mutations in *CARD10* on NF*κ*B signaling, apoptosis, and the development of POAG.

## Conflict of Interest

The authors declare that they have no conflict of interest.

## Supporting information


**Figure S1.** Chromatograms of all eight *CARD10* mutation carriers in the POAG cohort.
**Table S1.** PCR primers for validation of *CARD10* (NM_ 014550.3) variants by direct sequencing.
**Table S2.** List of genes found on GWAS to be associated with POAG or endophenotype.
**Table S3.** Summary of disease burden in genes near GWAS associated SNPs.
**Table S4.** 86 randomly selected genes (Set 1 of 5).
**Table S5.** 86 randomly selected genes (Set 2 of 5).
**Table S6.** 86 randomly selected genes (Set 3 of 5).
**Table S7.** 86 randomly selected genes (Set 4 of 5).
**Table S8.** 86 randomly selected genes (Set 5 of 5).

## References

[mgg3248-bib-0001] Axenovich, T. , I. Zorkoltseva , N. Belonogova , L. M. van Koolwijk , P. Borodin , A. Kirichenko , et al. 2011 Linkage and association analyses of glaucoma related traits in a large pedigree from a Dutch genetically isolated population. J. Med. Genet. 48:802–809. 22058429 10.1136/jmedgenet-2011-100436

[mgg3248-bib-0002] Bailey, J. N. , S. J. Loomis , J. H. Kang , R. R. Allingham , P. Gharahkhani , C. C. Khor , et al. 2016 Genome‐wide association analysis identifies TXNRD2, ATXN2 and FOXC1 as susceptibility loci for primary open‐angle glaucoma. Nat. Genet. 48:189–194. 26752265 10.1038/ng.3482 PMC4731307

[mgg3248-bib-0003] Burdon, K. P. , S. Macgregor , A. W. Hewitt , S. Sharma , G. Chidlow , R. A. Mills , et al. 2011 Genome‐wide association study identifies susceptibility loci for open angle glaucoma at TMCO1 and CDKN2B‐AS1. Nat. Genet. 43:574–578. 21532571 10.1038/ng.824

[mgg3248-bib-0004] Carnes, M. U. , Y. P. Liu , R. R. Allingham , B. T. Whigham , S. Havens , M. E. Garrett , et al. 2014 Discovery and functional annotation of SIX6 variants in primary open‐angle glaucoma. PLoS Genet. 10:e1004372. 24875647 10.1371/journal.pgen.1004372 PMC4038608

[mgg3248-bib-0005] Chen, Y. , Y. Lin , E. N. Vithana , L. Jia , X. Zuo , T. Y. Wong , et al. 2014 Common variants near ABCA1 and in PMM2 are associated with primary open‐angle glaucoma. Nat. Genet. 46:1115–1119. 25173107 10.1038/ng.3078

[mgg3248-bib-0006] Cowan, C. , C. K. Muraleedharan , J. J. 3rd O'Donnell , P. K. Singh , H. Lum , A. Kumar , et al. 2014 MicroRNA‐146 inhibits thrombin‐induced NF‐kappaB activation and subsequent inflammatory responses in human retinal endothelial cells. Invest. Ophthalmol. Vis. Sci. 55:4944–4951. 24985472 10.1167/iovs.13-13631

[mgg3248-bib-0007] DePristo, M. A. , E. Banks , R. Poplin , K. V. Garimella , J. R. Maguire , C. Hartl , et al. 2011 A framework for variation discovery and genotyping using next‐generation DNA sequencing data. Nat. Genet. 43:491–498. 21478889 10.1038/ng.806 PMC3083463

[mgg3248-bib-0008] Du, S. , L. Jia , Y. Zhang , L. Fang , X. Zhang , and Y. Fan . 2014 CARMA3 is upregulated in human pancreatic carcinoma, and its depletion inhibits tumor proliferation, migration, and invasion. Tumour Biol. 35:5965–5970. 24633921 10.1007/s13277-014-1791-6

[mgg3248-bib-0009] Escarcega, R. O. , S. Fuentes‐Alexandro , M. Garcia‐Carrasco , A. Gatica , and A. Zamora . 2007 The transcription factor nuclear factor‐kappa B and cancer. Clin. Oncol. (R. Coll. Radiol.) 19:154–161. 17355113 10.1016/j.clon.2006.11.013

[mgg3248-bib-0010] Estrada, K. , U. Styrkarsdottir , E. Evangelou , Y. H. Hsu , E. L. Duncan , E. E. Ntzani , et al. 2012 Genome‐wide meta‐analysis identifies 56 bone mineral density loci and reveals 14 loci associated with risk of fracture. Nat. Genet. 44:491–501. 22504420 10.1038/ng.2249 PMC3338864

[mgg3248-bib-0011] Fingert, J. H. 2011 Primary open‐angle glaucoma genes. Eye (London) 25:587–595. 10.1038/eye.2011.97 PMC3171270 21562585

[mgg3248-bib-0012] Foster, P. J. , R. Buhrmann , H. A. Quigley , and G. J. Johnson . 2002 The definition and classification of glaucoma in prevalence surveys. Br. J. Ophthalmol. 86:238–242. 11815354 10.1136/bjo.86.2.238 PMC1771026

[mgg3248-bib-0013] Gao, X. , W. J. Gauderman , Y. Liu , P. Marjoram , M. Torres , T. Haritunians , et al. 2013 A genome‐wide association study of central corneal thickness in Latinos. Invest. Ophthalmol. Vis. Sci. 54:2435–2443. 23493294 10.1167/iovs.13-11692 PMC3621577

[mgg3248-bib-0014] Gharahkhani, P. , K. P. Burdon , R. Fogarty , S. Sharma , A. W. Hewitt , S. Martin , et al. 2014 Common variants near ABCA1, AFAP1 and GMDS confer risk of primary open‐angle glaucoma. Nat. Genet. 46:1120–1125. 25173105 10.1038/ng.3079 PMC4177327

[mgg3248-bib-0015] Grabiner, B. C. , M. Blonska , P. C. Lin , Y. You , D. Wang , J. Sun , et al. 2007 CARMA3 deficiency abrogates G protein‐coupled receptor‐induced NF‐{kappa}B activation. Genes Dev. 21:984–996. 17438001 10.1101/gad.1502507 PMC1847715

[mgg3248-bib-0016] Healey, P. R. , and P. Mitchell . 1999 Optic disk size in open‐angle glaucoma: the Blue Mountains Eye Study. Am. J. Ophthalmol. 128:515–517. 10577600 10.1016/s0002-9394(99)00195-6

[mgg3248-bib-0017] Hoehn, R. , T. Zeller , V. J. Verhoeven , F. Grus , M. Adler , R. C. Wolfs , et al. 2012 Population‐based meta‐analysis in Caucasians confirms association with COL5A1 and ZNF469 but not COL8A2 with central corneal thickness. Hum. Genet. 131:1783–1793. 22814818 10.1007/s00439-012-1201-3

[mgg3248-bib-0018] Hollands, H. , D. Johnson , S. Hollands , D. L. Simel , D. Jinapriya , and S. Sharma . 2013 Do findings on routine examination identify patients at risk for primary open‐angle glaucoma? The rational clinical examination systematic review. JAMA 309:2035–2042. 23677315 10.1001/jama.2013.5099

[mgg3248-bib-0019] Hysi, P. G. , C. Y. Cheng , H. Springelkamp , S. Macgregor , J. N. Bailey , R. Wojciechowski , et al. 2014 Genome‐wide analysis of multi‐ancestry cohorts identifies new loci influencing intraocular pressure and susceptibility to glaucoma. Nat. Genet. 46:1126–1130. 25173106 10.1038/ng.3087 PMC4177225

[mgg3248-bib-0020] Jiang, X. , R. Varma , S. Wu , M. Torres , S. P. Azen , B. A. Francis , et al. 2012 Baseline risk factors that predict the development of open‐angle glaucoma in a population: the Los Angeles Latino Eye Study. Ophthalmology 119:2245–2253. 22796305 10.1016/j.ophtha.2012.05.030 PMC3474872

[mgg3248-bib-0021] Kaltschmidt, B. , D. Widera , and C. Kaltschmidt . 2005 Signaling via NF‐kappaB in the nervous system. Biochim. Biophys. Acta 1745:287–299. 15993497 10.1016/j.bbamcr.2005.05.009

[mgg3248-bib-0022] Khor, C. C. , W. D. Ramdas , E. N. Vithana , B. K. Cornes , X. Sim , W. T. Tay , et al. 2011 Genome‐wide association studies in Asians confirm the involvement of ATOH7 and TGFBR3, and further identify CARD10 as a novel locus influencing optic disc area. Hum. Mol. Genet. 20:1864–1872. 21307088 10.1093/hmg/ddr060

[mgg3248-bib-0023] Kitaoka, Y. , Y. Munemasa , T. Nakazawa , and S. Ueno . 2007 NMDA‐induced interleukin‐1beta expression is mediated by nuclear factor‐kappa B p65 in the retina. Brain Res. 1142:247–255. 17320050 10.1016/j.brainres.2007.01.097

[mgg3248-bib-0024] van Koolwijk, L. M. , W. D. Ramdas , M. K. Ikram , N. M. Jansonius , F. Pasutto , P. G. Hysi , et al. 2012 Common genetic determinants of intraocular pressure and primary open‐angle glaucoma. PLoS Genet. 8:e1002611. 22570627 10.1371/journal.pgen.1002611 PMC3342933

[mgg3248-bib-0025] Kwon, Y. H. , J. H. Fingert , M. H. Kuehn , and W. L. Alward . 2009 Primary open‐angle glaucoma. N. Engl. J. Med. 360:1113–1124. 19279343 10.1056/NEJMra0804630 PMC3700399

[mgg3248-bib-0026] Leske, M. C. , A. Heijl , M. Hussein , B. Bengtsson , L. Hyman , and E. Komaroff . 2003 Factors for glaucoma progression and the effect of treatment: the early manifest glaucoma trial. Arch. Ophthalmol. 121:48–56. 12523884 10.1001/archopht.121.1.48

[mgg3248-bib-0027] Li, Z. , L. Qu , Q. Dong , B. Huang , H. Li , Z. Tang , et al. 2012 Overexpression of CARMA3 in non‐small‐cell lung cancer is linked for tumor progression. PLoS ONE 7:e36903. 22615840 10.1371/journal.pone.0036903 PMC3352848

[mgg3248-bib-0028] Li, Z. , R. R. Allingham , M. Nakano , L. Jia , Y. Chen , Y. Ikeda , et al. 2015 A common variant near TGFBR3 is associated with primary open angle glaucoma. Hum. Mol. Genet. 24:3880–3892. 25861811 10.1093/hmg/ddv128 PMC4459396

[mgg3248-bib-0029] Lu, Y. , V. Vitart , K. P. Burdon , C. C. Khor , Y. Bykhovskaya , A. Mirshahi , et al. 2013 Genome‐wide association analyses identify multiple loci associated with central corneal thickness and keratoconus. Nat. Genet. 45:155–163. 23291589 10.1038/ng.2506 PMC3720123

[mgg3248-bib-0030] Macgregor, S. , A. W. Hewitt , P. G. Hysi , J. B. Ruddle , S. E. Medland , A. K. Henders , et al. 2010 Genome‐wide association identifies ATOH7 as a major gene determining human optic disc size. Hum. Mol. Genet. 19:2716–2724. 20395239 10.1093/hmg/ddq144 PMC2883339

[mgg3248-bib-0031] Mackey, D. A. , and A. W. Hewitt . 2014 Genome‐wide association study success in ophthalmology. Curr. Opin. Ophthalmol. 25:386–393. 25014751 10.1097/ICU.0000000000000090

[mgg3248-bib-0032] Man, X. , J. He , C. Kong , Y. Zhu , and Z. Zhang . 2014 Clinical significance and biological roles of CARMA3 in human bladder carcinoma. Tumour Biol. 35:4131–4136. 24443255 10.1007/s13277-013-1540-2

[mgg3248-bib-0033] Manolio, T. A. , F. S. Collins , N. J. Cox , D. B. Goldstein , L. A. Hindorff , D. J. Hunter , et al. 2009 Finding the missing heritability of complex diseases. Nature 461:747–753. 19812666 10.1038/nature08494 PMC2831613

[mgg3248-bib-0034] McAllister‐Lucas, L. M. , N. Inohara , P. C. Lucas , J. Ruland , A. Benito , Q. Li , et al. 2001 Bimp1, a MAGUK family member linking protein kinase C activation to Bcl10‐mediated NF‐kappaB induction. J. Biol. Chem. 276:30589–30597. 11387339 10.1074/jbc.M103824200

[mgg3248-bib-0035] McKenna, A. , M. Hanna , E. Banks , A. Sivachenko , K. Cibulskis , A. Kernytsky , et al. 2010 The Genome Analysis Toolkit: a MapReduce framework for analyzing next‐generation DNA sequencing data. Genome Res. 20:1297–1303. 20644199 10.1101/gr.107524.110 PMC2928508

[mgg3248-bib-0036] Meguro, A. , H. Inoko , M. Ota , N. Mizuki , and S. Bahram . 2010 Genome‐wide association study of normal tension glaucoma: common variants in SRBD1 and ELOVL5 contribute to disease susceptibility. Ophthalmology 117:1331–1338. e5. 20363506 10.1016/j.ophtha.2009.12.001

[mgg3248-bib-0037] Miao, Z. , T. Zhao , Z. Wang , Y. Xu , Y. Song , J. Wu , et al. 2012 CARMA3 is overexpressed in colon cancer and regulates NF‐kappaB activity and cyclin D1 expression. Biochem. Biophys. Res. Commun. 425:781–787. 22884800 10.1016/j.bbrc.2012.07.152

[mgg3248-bib-0038] Miglior, S. , N. Pfeiffer , V. Torri , T. Zeyen , J. Cunha‐Vaz , and I. Adamsons . 2007 Predictive factors for open‐angle glaucoma among patients with ocular hypertension in the European Glaucoma Prevention Study. Ophthalmology 114:3–9. 17070596 10.1016/j.ophtha.2006.05.075

[mgg3248-bib-0039] Nag, A. , C. Venturini , K. S. Small , T. L. Young , A. C. Viswanathan , D. A. Mackey , et al. 2014 A genome‐wide association study of intra‐ocular pressure suggests a novel association in the gene FAM125B in the TwinsUK cohort. Hum. Mol. Genet. 23:3343–3348. 24518671 10.1093/hmg/ddu050 PMC4030784

[mgg3248-bib-0040] Nakano, M. , Y. Ikeda , Y. Tokuda , M. Fuwa , N. Omi , M. Ueno , et al. 2012 Common variants in CDKN2B‐AS1 associated with optic‐nerve vulnerability of glaucoma identified by genome‐wide association studies in Japanese. PLoS ONE 7:e33389. 22428042 10.1371/journal.pone.0033389 PMC3299784

[mgg3248-bib-0041] Osman, W. , S. K. Low , A. Takahashi , M. Kubo , and Y. Nakamura . 2012 A genome‐wide association study in the Japanese population confirms 9p21 and 14q23 as susceptibility loci for primary open angle glaucoma. Hum. Mol. Genet. 21:2836–2842. 22419738 10.1093/hmg/dds103

[mgg3248-bib-0042] Ozel, A. B. , S. E. Moroi , D. M. Reed , M. Nika , C. M. Schmidt , S. Akbari , et al. 2014 Genome‐wide association study and meta‐analysis of intraocular pressure. Hum. Genet. 133:41–57. 24002674 10.1007/s00439-013-1349-5 PMC3982323

[mgg3248-bib-0043] Pascolini, D. , and S. P. Mariotti . 2012 Global estimates of visual impairment: 2010. Br. J. Ophthalmol. 96:614–618. 22133988 10.1136/bjophthalmol-2011-300539

[mgg3248-bib-0044] Philomenadin, F. S. , R. Asokan , N. Viswanathan , R. George , V. Lingam , and S. Sarangapani . 2015 Genetic association of SNPs near ATOH7, CARD10, CDKN2B, CDC7 and SIX1/SIX6 with the endophenotypes of primary open angle glaucoma in Indian population. PLoS ONE 10:e0119703. 25798827 10.1371/journal.pone.0119703 PMC4370747

[mgg3248-bib-0045] Ramdas, W. D. , L. M. van Koolwijk , M. K. Ikram , N. M. Jansonius , P. T. de Jong , A. A. Bergen , et al. 2010 A genome‐wide association study of optic disc parameters. PLoS Genet. 6:e1000978. 20548946 10.1371/journal.pgen.1000978 PMC2883590

[mgg3248-bib-0046] Ramdas, W. D. , L. M. van Koolwijk , H. G. Lemij , F. Pasutto , A. J. Cree , G. Thorleifsson , et al. 2011 Common genetic variants associated with open‐angle glaucoma. Hum. Mol. Genet. 20:2464–2471. 21427129 10.1093/hmg/ddr120

[mgg3248-bib-0047] Rau, C. S. , J. C. Yang , Y. C. Chen , C. J. Wu , T. H. Lu , S. L. Tzeng , et al. 2014 Lipopolysaccharide‐induced microRNA‐146a targets CARD10 and regulates angiogenesis in human umbilical vein endothelial cells. Toxicol. Sci. 140:315–326. 24863965 10.1093/toxsci/kfu097

[mgg3248-bib-0048] Scudiero, I. , P. Vito , and R. Stilo . 2014 The three CARMA sisters: so different, so similar: a portrait of the three CARMA proteins and their involvement in human disorders. J. Cell. Physiol. 229:990–997. 24375035 10.1002/jcp.24543

[mgg3248-bib-0049] Siu, H. , Y. Zhu , L. Jin , and M. Xiong . 2011 Implication of next‐generation sequencing on association studies. BMC Genom. 12:322. 10.1186/1471-2164-12-322 PMC3148210 21682891

[mgg3248-bib-0050] Souzeau, E. , I. Goldberg , P. R. Healey , R. A. Mills , J. Landers , S. L. Graham , et al. 2012 Australian and New Zealand registry of advanced glaucoma: methodology and recruitment. Clin. Exp. Ophthalmol. 40:569–575. 22171965 10.1111/j.1442-9071.2011.02742.x

[mgg3248-bib-0051] Springelkamp, H. , R. Hohn , A. Mishra , P. G. Hysi , C. C. Khor , S. J. Loomis , et al. 2014 Meta‐analysis of genome‐wide association studies identifies novel loci that influence cupping and the glaucomatous process. Nat. Commun. 5:4883. 25241763 10.1038/ncomms5883 PMC4199103

[mgg3248-bib-0052] Springelkamp, H. , A. Mishra , P. G. Hysi , P. Gharahkhani , R. Hohn , C. C. Khor , et al. 2015 Meta‐analysis of genome‐wide association studies identifies novel loci associated with optic disc morphology. Genet. Epidemiol. 39:207–216. 25631615 10.1002/gepi.21886 PMC4480365

[mgg3248-bib-0053] Strange, A. , C. Bellenguez , X. Sim , R. Luben , P. G. Hysi , W. D. Ramdas , et al. 2013 Genome‐wide association study of intraocular pressure identifies the GLCCI1/ICA1 region as a glaucoma susceptibility locus. Hum. Mol. Genet. 22:4653–4660. 23836780 10.1093/hmg/ddt293 PMC3904806

[mgg3248-bib-0054] Takamoto, M. , T. Kaburaki , A. Mabuchi , M. Araie , S. Amano , M. Aihara , et al. 2012 Common variants on chromosome 9p21 are associated with normal tension glaucoma. PLoS ONE 7:e40107. 22792221 10.1371/journal.pone.0040107 PMC3390321

[mgg3248-bib-0055] Tham, Y. C. , X. Li , T. Y. Wong , H. A. Quigley , T. Aung , and C. Y. Cheng . 2014 Global prevalence of glaucoma and projections of glaucoma burden through 2040: a systematic review and meta‐analysis. Ophthalmology 121:2081–2090. 24974815 10.1016/j.ophtha.2014.05.013

[mgg3248-bib-0056] Thorleifsson, G. , G. B. Walters , A. W. Hewitt , G. Masson , A. Helgason , A. DeWan , et al. 2010 Common variants near CAV1 and CAV2 are associated with primary open‐angle glaucoma. Nat. Genet. 42:906–909. 20835238 10.1038/ng.661 PMC3222888

[mgg3248-bib-0057] Ulmer, M. , J. Li , B. L. Yaspan , A. B. Ozel , J. E. Richards , S. E. Moroi , et al. 2012 Genome‐wide analysis of central corneal thickness in primary open‐angle glaucoma cases in the NEIGHBOR and GLAUGEN consortia. Invest. Ophthalmol. Vis. Sci. 53:4468–4474. 22661486 10.1167/iovs.12-9784 PMC3394688

[mgg3248-bib-0058] Van der Auwera, G. A. , M. O. Carneiro , C. Hartl , R. Poplin , G. Del Angel , A. Levy‐Moonshine , et al. 2013 From FastQ data to high confidence variant calls: the genome analysis toolkit best practices pipeline. Curr. Protoc. Bioinformatics 11:11 10 1–11 10 33. 10.1002/0471250953.bi1110s43 PMC4243306 25431634

[mgg3248-bib-0059] Vitart, V. , G. Bencic , C. Hayward , J. Skunca Herman , J. Huffman , S. Campbell , et al. 2010 New loci associated with central cornea thickness include COL5A1, AKAP13 and AVGR8. Hum. Mol. Genet. 19:4304–4311. 20719862 10.1093/hmg/ddq349

[mgg3248-bib-0060] Vithana, E. N. , T. Aung , C. C. Khor , B. K. Cornes , W. T. Tay , X. Sim , et al. 2011 Collagen‐related genes influence the glaucoma risk factor, central corneal thickness. Hum. Mol. Genet. 20:649–658. 21098505 10.1093/hmg/ddq511

[mgg3248-bib-0061] Wang, L. , Y. Guo , W. J. Huang , X. Ke , J. L. Poyet , G. A. Manji , et al. 2001 Card10 is a novel caspase recruitment domain/membrane‐associated guanylate kinase family member that interacts with BCL10 and activates NF‐kappa B. J. Biol. Chem. 276:21405–21409. 11259443 10.1074/jbc.M102488200

[mgg3248-bib-0062] Wang, K. , M. Li , and H. Hakonarson . 2010 ANNOVAR: functional annotation of genetic variants from high‐throughput sequencing data. Nucleic Acids Res. 38:e164. 20601685 10.1093/nar/gkq603 PMC2938201

[mgg3248-bib-0063] Wiggs, J. L. , J. H. Kang , B. L. Yaspan , D. B. Mirel , C. Laurie , A. Crenshaw , et al. 2011 Common variants near CAV1 and CAV2 are associated with primary open‐angle glaucoma in Caucasians from the USA. Hum. Mol. Genet. 20:4707–4713. 21873608 10.1093/hmg/ddr382 PMC3209825

[mgg3248-bib-0064] Wiggs, J. L. , B. L. Yaspan , M. A. Hauser , J. H. Kang , R. R. Allingham , L. M. Olson , et al. 2012 Common variants at 9p21 and 8q22 are associated with increased susceptibility to optic nerve degeneration in glaucoma. PLoS Genet. 8:e1002654. 22570617 10.1371/journal.pgen.1002654 PMC3343074

[mgg3248-bib-0065] Wolfs, R. C. , C. C. Klaver , R. S. Ramrattan , C. M. van Duijn , A. Hofman , and P. T. de Jong . 1998 Genetic risk of primary open‐angle glaucoma. Population‐based familial aggregation study. Arch. Ophthalmol. 116:1640–1645. 9869795 10.1001/archopht.116.12.1640

[mgg3248-bib-0066] Wu, G. L. , J. L. Yuan , X. D. Huang , J. F. Rong , L. X. Zhang , Y. P. Liu , et al. 2013 Evaluating the expression of CARMA3 as a prognostic tumor marker in renal cell carcinoma. Tumour Biol. 34:3431–3435. 23771851 10.1007/s13277-013-0917-6

[mgg3248-bib-0067] Xie, C. , Y. Han , L. Fu , Q. Li , X. Qiu , and E. Wang . 2014 Overexpression of CARMA3 is associated with advanced tumor stage, cell cycle progression, and cisplatin resistance in human epithelial ovarian cancer. Tumour Biol. 35:7957–7964. 24833094 10.1007/s13277-014-2070-2

[mgg3248-bib-0068] Zhao, T. , Z. Miao , Z. Wang , Y. Xu , J. Wu , X. Liu , et al. 2013 CARMA3 overexpression accelerates cell proliferation and inhibits paclitaxel‐induced apoptosis through NF‐kappaB regulation in breast cancer cells. Tumour Biol. 34:3041–3047. 23708960 10.1007/s13277-013-0869-x

