# Supplementary material for: Rare variants in optic disc area gene CARD10 enriched in primary open‐angle glaucoma
Source: Mol Genet Genomic Med. 2016 Oct 3;4(6):624–33. doi: 10.1002/mgg3.248 (PMC5118207; doi:10.1002/mgg3.248)
Supplement: Supplementary file 1 — Figure S1. Chromatograms of all eight CARD10 mutation carriers in the POAG cohort. Table S1. PCR primers for validation of CARD10 (NM_ 014550.3) variants by direct sequencing. Table S2. List of genes found on GWAS to be associated with POAG or endophenotype. Table S3. Summary of disease burden in genes near GWAS associated SNPs. Table S4. 86 randomly selected genes (Set 1 of 5). Table S5. 86 randomly selected genes (Set 2 of 5). Table S6. 86 randomly selected genes (Set 3 of 5). Table S7. 86 randomly selected genes (Set 4 of 5). Table S8. 86 randomly selected genes (Set 5 of 5). [file MGG3-4-624-s001.docx]

**Supplementary Table 1**: PCR primers for validation of *CARD10* variants by direct sequencing. PCR optimized at 62-64°C

| **Position** | **Exon** | **cDNA change** | **Residue change** | **Forward Primer (5’>3’)** | **Reverse Primer (5’>3’)** |
| --- | --- | --- | --- | --- | --- |
| chr22:37912044 | 3 | c.635G>A | p.Arg212His | tccagaatttccctctagtgttt | ttatccacgtcaaagagccag |
| chr22:37904616 | 5 | c.983C>T | p.Ala328Val | tgtccccttccttcccacac | gtgcctccgtcaacatctga |
| chr22:37904575 | 5 | c.1024G>A | p.Val342Met | tgtccccttccttcccacac | gtgcctccgtcaacatctga |
| chr22:37902372 | 7 | c.1210C>T | p.Arg404Trp | agctgccattctccttactgt | ttcaagttcccgcccctaac |
| chr22:37888801 | 17 | c.2485C>T | p.Arg829Trp | gttttggggtatcgacgagc | acaggagggaaaggacttgg |

**Supplementary Table 2**: List of genes found on GWAS to be associated with POAG or endophenotype. **Bold** = Genes analysed in this study. CCT = Central corneal thickness, ODA = Optic disc area, CA = Cup area, IOP = Intraocular pressure, NRR = Neuroretinal rim, VCDR = Vertical cup-to-disc ratio, NTG = Normal-tension glaucoma.

| **Locus** | **Genes** | **Top SNP** | **Phenotype** | **Populations** | **Publication** |
| --- | --- | --- | --- | --- | --- |
| 1p22 | ***CDC7, TGFBR3*** | rs1192419 | ODA | Dutch | ([Axenovich et al., 2011](#_ENREF_1)) |
|  |  | - | ODA | Asian | ([Khor et al., 2011](#_ENREF_9)) |
|  |  | rs1192415 | ODA | Caucasian | ([Ramdas et al., 2010](#_ENREF_18)) |
|  |  | rs1192415 | POAG | Asian, Caucasian, African | ([Li et al., 2015](#_ENREF_10)) |
|  |  | rs4658101 | VCDR | Asian, Caucasian | ([Springelkamp et al., 2014](#_ENREF_20)) |
| 1p34.2 | ***COL8A2*** | rs96067 | CCT | Caucasian | ([Lu et al., 2013](#_ENREF_11)) |
|  |  | rs96067 | CCT | Malay, Indian | ([Vithana et al., 2011](#_ENREF_28)) |
| 1p36 | ***RERE*** | rs2252865 | NRR | Dutch | ([Axenovich et al., 2011](#_ENREF_1)) |
|  |  | rs2252865 | VCDR | Dutch | ([Axenovich et al., 2011](#_ENREF_1)) |
|  |  | rs301801 | VCDR | Asian, Caucasian | ([Springelkamp et al., 2014](#_ENREF_20)) |
| 1p36.1 | ***DHRS3*** | rs3924048 | CA | Asian, Caucasian | ([Springelkamp et al., 2015](#_ENREF_21)) |
| 1q23 | ***F5*** | rs12406092 | ODA | Asian, Caucasian | ([Springelkamp et al., 2015](#_ENREF_21)) |
| 1q24.1 | ***TMCO1*** | rs7555523 | IOP | Asian, Caucasian | ([Hysi et al., 2014](#_ENREF_8)) |
|  |  | rs7518099 | IOP | Caucasian | ([Ozel et al., 2014](#_ENREF_17)) |
|  |  | rs7555523 | IOP | Caucasian | ([van Koolwijk et al., 2012](#_ENREF_26)) |
|  |  | rs4656461 | POAG | Caucasian | ([Burdon et al., 2011](#_ENREF_3)) |
|  |  | rs7555523 | POAG | Asian, Caucasian | ([Hysi et al., 2014](#_ENREF_8)) |
|  |  | rs7555523 | POAG | Caucasian | ([van Koolwijk et al., 2012](#_ENREF_26)) |
| 1q42.11 | ***CDC42BPA*** | rs6671926 | ODA | Asian, Caucasian | ([Springelkamp et al., 2015](#_ENREF_21)) |
| 2p16 | ***EFEMP1*** | rs1346786 | CA | Asian, Caucasian | ([Springelkamp et al., 2015](#_ENREF_21)) |
| 2p21 | ***SRBD1*** | rs3213787 | NTG | Japanese | ([Meguro et al., 2010](#_ENREF_13)) |
| 2p24.3 | ***TRIB2*** | rs2113818 | CA | Asian, Caucasian | ([Springelkamp et al., 2015](#_ENREF_21)) |
| 2q35 | ***USP37*** | rs10189064 | CCT | Caucasian | ([Lu et al., 2013](#_ENREF_11)) |
| 2q35 | *DIRC3* | rs1549733 | ODA | Asian, Caucasian | ([Springelkamp et al., 2015](#_ENREF_21)) |
| 2q36 | ***COL4A3*** | rs7606754 | CCT | Asian, Caucasian | ([Lu et al., 2013](#_ENREF_11)) |
| 3p14.3 | ***FLNB*** | rs6764184 | CA | Asian, Caucasian | ([Springelkamp et al., 2015](#_ENREF_21)) |
| 3p24.2 | ***RARB*** | rs11129176 | ODA | Asian, Caucasian | ([Springelkamp et al., 2015](#_ENREF_21)) |
| 3q11.2 | ***GPR15*** | rs3749260 | CCT | Caucasian | ([Lu et al., 2013](#_ENREF_11)) |
| 3q12 | ***COL8A1*** | rs2623325 | VCDR | Asian, Caucasian | ([Springelkamp et al., 2014](#_ENREF_20)) |
| 3q12.2 | ***ABI3BP*** | rs9860250 | ODA | Asian, Caucasian | ([Springelkamp et al., 2015](#_ENREF_21)) |
| 3q25.31 | ***TIPARP*** | rs9822953 | CCT | Caucasian | ([Lu et al., 2013](#_ENREF_11)) |
| 3q26.31 | ***FNDC3B*** | rs4894535 | CCT | Asian, Caucasian | ([Lu et al., 2013](#_ENREF_11)) |
|  |  | rs6445055 | IOP | Asian, Caucasian | ([Hysi et al., 2014](#_ENREF_8)) |
|  |  | rs4894535 | POAG | Asian, Caucasian | ([Lu et al., 2013](#_ENREF_11)) |
| 3q26.32 | ***KCNMB2,*** *TBL1XR1* | rs7620503 | CCT | Asian, Caucasian | ([Lu et al., 2013](#_ENREF_11)) |
| 4p16.1 | ***AFAP1*** | rs4619890 | POAG | Caucasian | ([Gharahkhani et al., 2014](#_ENREF_6)) |
| 4q31 | *NR3C2* | rs3931397 | CCT | Asian, Caucasian | ([Lu et al., 2013](#_ENREF_11)) |
| 5q12 | ***ADAMTS6, CWC27*** | rs1117707 | CCT | Caucasian | ([Lu et al., 2013](#_ENREF_11)) |
| 5q35.1 | ***DUSP1*** | rs17658229 | VCDR | Asian, Caucasian | ([Springelkamp et al., 2014](#_ENREF_20)) |
| 6p12.1 | ***ELOVL5*** | rs735860 | NTG | Japanese | ([Meguro et al., 2010](#_ENREF_13)) |
| 6p25.3 | ***GMDS*** | rs11969985 | POAG | Caucasian | ([Gharahkhani et al., 2014](#_ENREF_6)) |
| 6p25.3 | ***FOXC1*** | rs2745572 | POAG | Asian, Caucasian | ([Bailey et al., 2016](#_ENREF_2)) |
| 6p25.3 | ***EXOC2*** | rs17756712 | VCDR | Asian, Caucasian | ([Springelkamp et al., 2014](#_ENREF_20)) |
| 6q14 | ***FAM46A*** | rs1538138 | CCT | Caucasian | ([Lu et al., 2013](#_ENREF_11)) |
| 6q14.1 | ***IBTK*** | rs1538138 | CCT | Caucasian | ([Lu et al., 2013](#_ENREF_11)) |
| 6q22.31 | ***HSF2*** | rs868153 | VCDR | Asian, Caucasian | ([Springelkamp et al., 2014](#_ENREF_20)) |
| 7p21 | ***GLCCI1***, ***ICA1*** | rs59072263 | IOP | Caucasian | ([Strange et al., 2013](#_ENREF_22)) |
| 7q11.21 | *C7orf42* | rs4718428 | CCT | Caucasian | ([Lu et al., 2013](#_ENREF_11)) |
| 7q11.21 | ***VKORC1L1*** | rs11763147 | CCT | Asian, Caucasian | ([Lu et al., 2013](#_ENREF_11)) |
| 7q31 | ***CAV1, CAV2*** | rs10258482 | IOP | Asian, Caucasian | ([Hysi et al., 2014](#_ENREF_8)) |
|  |  | rs10258482 | POAG | Asian, Caucasian | ([Hysi et al., 2014](#_ENREF_8)) |
|  |  | rs4236601 | POAG | Caucasian | ([Thorleifsson et al., 2010](#_ENREF_24)) |
|  |  | rs1052990 | POAG | Caucasian | ([Wiggs et al., 2011](#_ENREF_29)) |
| 8q21.3 | ***DCAF4L2*** | rs9969524 | ODA | Asian, Caucasian | ([Springelkamp et al., 2015](#_ENREF_21)) |
| 9p22.3 | ***NFIB*** | rs1324183 | CCT | Caucasian | ([Lu et al., 2013](#_ENREF_11)) |
| 9p23 | ***MPDZ*** | rs1324183 | CCT | Caucasian | ([Lu et al., 2013](#_ENREF_11)) |
| 9q21 | ***CDKN2A,*** | rs523096 | NTG | Japanese | ([Takamoto et al., 2012](#_ENREF_23)) |
|  | ***CDKN2B,*** | rs2157719 | NTG | Caucasian | ([Wiggs et al., 2012](#_ENREF_30)) |
|  | *CDKN2B-AS1* | rs4977756 | POAG | Caucasian | ([Burdon et al., 2011](#_ENREF_3)) |
|  |  | rs2157719 | POAG | Asian, Caucasian, African | ([Li et al., 2015](#_ENREF_10)) |
|  |  | rs7865618 | POAG | Japanese | ([Nakano et al., 2012](#_ENREF_15)) |
|  |  | rs1063192 | POAG | Japanese | ([Osman et al., 2012](#_ENREF_16)) |
|  |  | rs1063192 | POAG | Caucasian | ([Ramdas et al., 2011](#_ENREF_19)) |
|  |  | rs2157719 | POAG | Caucasian | ([Wiggs et al., 2012](#_ENREF_30)) |
|  |  | rs1063192 | VCDR | Caucasian | ([Ramdas et al., 2010](#_ENREF_18)) |
|  |  | rs7865618 | VCDR | Asian, Caucasian | ([Springelkamp et al., 2014](#_ENREF_20)) |
| 9q31.1 | ***ABCA1*** | rs2472493 | IOP | Asian, Caucasian | ([Hysi et al., 2014](#_ENREF_8)) |
|  |  | rs2487032 | POAG | Chinese | ([Chen et al., 2014](#_ENREF_4)) |
|  |  | rs2472493 | POAG | Caucasian | ([Gharahkhani et al., 2014](#_ENREF_6)) |
|  |  | rs2472493 | POAG | Asian, Caucasian | ([Hysi et al., 2014](#_ENREF_8)) |
| 9q31.3 | ***LPAR1*** | rs1007000 | CCT | Asian, Caucasian | ([Lu et al., 2013](#_ENREF_11)) |
| 9q33.3 | ***FAM125B*** | rs2286885 | IOP | Caucasian | ([Nag et al., 2014](#_ENREF_14)) |
| 9q34.2 | *ABO* | rs8176743 | IOP | Asian, Caucasian | ([Hysi et al., 2014](#_ENREF_8)) |
| 9q34.2 | ***COL5A1*** | rs3118515 | CCT | Latino | ([Gao et al., 2013](#_ENREF_5)) |
|  | ***RXRA*** | rs1536482 | CCT | Croatian, Scottish | ([Vitart et al., 2010](#_ENREF_27)) |
|  |  | rs4842044 | CCT | Malay, Indian | ([Vithana et al., 2011](#_ENREF_28)) |
|  |  | rs3132306 | CCT | Caucasian | ([Hoehn et al., 2012](#_ENREF_7)) |
|  |  | rs3118520 | CCT | Caucasian | ([Lu et al., 2013](#_ENREF_11)) |
| 9q34.3 | ***LCN12,*** *PTGDS* | rs11145951 | CCT | Caucasian | ([Lu et al., 2013](#_ENREF_11)) |
| 10q21 | ***ATOH7*** | rs1900005 | ODA | Dutch | ([Axenovich et al., 2011](#_ENREF_1)) |
|  |  | - | ODA | Asian | ([Khor et al., 2011](#_ENREF_9)) |
|  |  | rs3858145 | ODA | Caucasian | ([Macgregor et al., 2010](#_ENREF_12)) |
|  |  | rs1900004 | ODA | Caucasian | ([Ramdas et al., 2010](#_ENREF_18)) |
|  |  | rs1900004 | POAG | Caucasian | ([Ramdas et al., 2011](#_ENREF_19)) |
|  |  | rs1900005 | VCDR | Asian, Caucasian | ([Springelkamp et al., 2014](#_ENREF_20)) |
| 10q21.2 | ***ARID5B*** | rs7090871 | CCT | Asian, Caucasian | ([Lu et al., 2013](#_ENREF_11)) |
| 10q23 | ***PLCE1*** | rs7072574 | VCDR | Asian, Caucasian | ([Springelkamp et al., 2014](#_ENREF_20)) |
| 11p11.2 | ***RAPSN*** | rs12419342 | IOP | Asian, Caucasian | ([Hysi et al., 2014](#_ENREF_8)) |
|  | ***NUP160*** | rs747782 | IOP | Asian, Caucasian | ([Hysi et al., 2014](#_ENREF_8)) |
|  | ***PTPRJ*** | rs1681630 | IOP | Asian, Caucasian | ([Hysi et al., 2014](#_ENREF_8)) |
| 11p13 | ***ELP4*** | rs11031436 | ODA | Asian, Caucasian | ([Springelkamp et al., 2015](#_ENREF_21)) |
| 11q13.1 | ***SSSCA1*** | rs1346 | VCDR | Asian, Caucasian | ([Springelkamp et al., 2014](#_ENREF_20)) |
| 11q23.1 | ***ARHGAP20****,* *POU2AF1* | rs4938174 | CCT | Asian, Caucasian | ([Lu et al., 2013](#_ENREF_11)) |
| 11q25 | ***ADAMTS8*** | rs4936099 | VCDR | Asian, Caucasian | ([Springelkamp et al., 2014](#_ENREF_20)) |
| 12q13.11 | ***RPAP3*** | rs11168187 | VCDR | Asian, Caucasian | ([Springelkamp et al., 2014](#_ENREF_20)) |
| 12q21.31 | ***TMTC2*** | rs10862688 | VCDR | Asian, Caucasian | ([Springelkamp et al., 2014](#_ENREF_20)) |
|  |  | rs1511589 | ODA | Asian, Caucasian | ([Springelkamp et al., 2015](#_ENREF_21)) |
| 12q23.3 | ***GLT8D2*** | rs1564892 | CCT | Asian, Caucasian | ([Lu et al., 2013](#_ENREF_11)) |
| 12q24.1 | ***ATXN2*** | rs7137828 | POAG | Asian, Caucasian | ([Bailey et al., 2016](#_ENREF_2)) |
| 12q24.31 | ***FAM101A*** | rs10846617 | CA | Asian, Caucasian | ([Springelkamp et al., 2015](#_ENREF_21)) |
| 13q11 | ***FGF9, SGCG*** | rs1034200 | CCT | Caucasian | ([Lu et al., 2013](#_ENREF_11)) |
| 13q12.11 | *AVGR8* | rs1034200 | CCT | Croatian, Scottish | ([Vitart et al., 2010](#_ENREF_27)) |
| 13q14.11 | ***FOXO1*** | rs2755237 | CCT | Croatian, Scottish | ([Vitart et al., 2010](#_ENREF_27)) |
|  |  | rs2721051 | CCT | Latino | ([Gao et al., 2013](#_ENREF_5)) |
|  |  | rs2755237 | CCT | Caucasian | ([Lu et al., 2013](#_ENREF_11)) |
|  |  | rs2721051 | CCT | Caucasian | ([Lu et al., 2013](#_ENREF_11)) |
| 14q22 | *SIX1,* ***SIX6*** | rs10483727 | POAG | Japanese | ([Osman et al., 2012](#_ENREF_16)) |
|  |  | rs10483727 | POAG | Caucasian | ([Ramdas et al., 2011](#_ENREF_19)) |
|  |  | rs10483727 | POAG | Caucasian | ([Wiggs et al., 2012](#_ENREF_30)) |
|  |  | rs10483727 | VCDR | Caucasian | ([Ramdas et al., 2010](#_ENREF_18)) |
|  |  | rs4901977 | VCDR | Asian, Caucasian | ([Springelkamp et al., 2014](#_ENREF_20)) |
| 14q22.2 | ***DDHD1, BMP4*** | rs10130556 | CA | Asian, Caucasian | ([Springelkamp et al., 2015](#_ENREF_21)) |
| 15q13 | ***TJP1*** | rs785422 | CCT | Caucasian | ([Lu et al., 2013](#_ENREF_11)) |
| 15q22.33 | *SMAD3* | rs12913547 | CCT | Asian, Caucasian | ([Lu et al., 2013](#_ENREF_11)) |
| 15q25.3 | ***AKAP13*** | rs6496932 | CCT | Caucasian | ([Lu et al., 2013](#_ENREF_11)) |
|  |  | rs6496932 | CCT | Croatian, Scottish | ([Vitart et al., 2010](#_ENREF_27)) |
| 15q26 | *NR2F2* | rs8034595 | ODA | Asian, Caucasian | ([Springelkamp et al., 2015](#_ENREF_21)) |
| 15q26.3 | ***CHSY1*** | rs752092 | CCT | Caucasian | ([Lu et al., 2013](#_ENREF_11)) |
| 15q26.3 | ***LRRK1*** | rs2034809 | CCT | Caucasian | ([Lu et al., 2013](#_ENREF_11)) |
| 15q26.3 | ***ASB7*** | rs11247230 | CA | Asian, Caucasian | ([Springelkamp et al., 2015](#_ENREF_21)) |
| 16p13.2 | ***PMM2*** | rs3785176 | POAG | Chinese | ([Chen et al., 2014](#_ENREF_4)) |
| 16q12.1 | ***SALL1*** | rs1362756 | ODA | Caucasian | ([Ramdas et al., 2010](#_ENREF_18)) |
|  |  | rs1345467 | VCDR | Asian, Caucasian | ([Springelkamp et al., 2014](#_ENREF_20)) |
| 16q24.2 | ***BANP*** | rs9938149 | CCT | Latino | ([Gao et al., 2013](#_ENREF_5)) |
|  | ***ZNF469*** | rs9938149 | CCT | Caucasian | ([Hoehn et al., 2012](#_ENREF_7)) |
|  |  | rs12447690 | CCT | Caucasian | ([Lu et al., 2013](#_ENREF_11)) |
|  |  | rs6540223 | CCT | Caucasian | ([Lu et al., 2013](#_ENREF_11)) |
|  |  | rs12447690 | CCT | Caucasian | ([Ulmer et al., 2012](#_ENREF_25)) |
|  |  | rs12447690 | CCT | Croatian, Scottish | ([Vitart et al., 2010](#_ENREF_27)) |
|  |  | rs9938149 | CCT | Malay, Indian | ([Vithana et al., 2011](#_ENREF_28)) |
| 17p12 | ***HS3ST3B1,*** *PMP22* | rs2323457 | CCT | Caucasian | ([Lu et al., 2013](#_ENREF_11)) |
| 17p13.1 | *GAS7* | rs9913911 | IOP | Asian, Caucasian | ([Hysi et al., 2014](#_ENREF_8)) |
|  |  | rs11656696 | IOP | Caucasian | ([van Koolwijk et al., 2012](#_ENREF_26)) |
|  |  | rs9913911 | POAG | Asian, Caucasian | ([Hysi et al., 2014](#_ENREF_8)) |
|  |  | rs11656696 | POAG | Caucasian | ([van Koolwijk et al., 2012](#_ENREF_26)) |
| 17q21.32 | ***KPNB1*** | rs11870935 | CA | Asian, Caucasian | ([Springelkamp et al., 2015](#_ENREF_21)) |
| 17q23.2 | ***BCAS3*** | rs11651885 | CA | Asian, Caucasian | ([Springelkamp et al., 2015](#_ENREF_21)) |
| 20p12 | *BMP2* | rs6054374 | VCDR | Asian, Caucasian | ([Springelkamp et al., 2014](#_ENREF_20)) |
| 22q11.21 | ***TXNRD2*** | rs35934224 | POAG | Asian, Caucasian | ([Bailey et al., 2016](#_ENREF_2)) |
| 22q12.2 | ***HORMAD2*** | rs2412970 | ODA | Asian, Caucasian | ([Springelkamp et al., 2015](#_ENREF_21)) |
| 22q13.1 | ***CARD10*** | rs9607469 | ODA | Asian, Caucasian | ([Khor et al., 2011](#_ENREF_9)) |
|  |  | rs5756813 | VCDR | Asian, Caucasian | ([Springelkamp et al., 2014](#_ENREF_20)) |
| 22q13.1 | ***TRIOBP*** | rs5756813 | CA | Asian, Caucasian | ([Springelkamp et al., 2015](#_ENREF_21)) |
| 22q21.1 | ***CHEK2*** | rs1547014 | VCDR | Asian, Caucasian | ([Springelkamp et al., 2014](#_ENREF_20)) |

**Supplementary Table 3**: Summary of disease burden in genes near GWAS associated SNPs.

| **Gene** | **POAG count** | **Control count** | **Odds ratio** | **Fisher's p value** | **Bonferroni corrected p** |
| --- | --- | --- | --- | --- | --- |
| *ABCA1* | 2 | 16 | 0.731785 | 1 | 1 |
| *ABI3BP* | 0 | 2 | 0 | 1 | 1 |
| *ADAMTS6* | 1 | 3 | 1.951277 | 0.4685225 | 1 |
| *ADAMTS8* | 6 | 41 | 0.846902 | 0.8371469 | 1 |
| *AFAP1* | 1 | 15 | 0.38574 | 0.4935098 | 1 |
| *AKAP13* | 12 | 42 | 1.637602 | 0.1739341 | 1 |
| *ARHGAP20* | 1 | 4 | 1.463904 | 0.5459818 | 1 |
| *ARID5B* | 1 | 8 | 0.731856 | 1 | 1 |
| *ASB7* | 0 | 2 | 0 | 1 | 1 |
| *ATOH7* | 0 | 1 | 0 | 1 | 1 |
| *ATXN2* | 3 | 14 | 1.142475 | 0.7419943 | 1 |
| *BANP* | 3 | 3 | 5.353832 | 0.0542457 | 1 |
| *BCAS3* | 1 | 7 | 0.836516 | 1 | 1 |
| *BMP4* | 0 | 10 | 0 | 0.3748709 | 1 |
| *CARD10* | 8 | 3 | 13.19481 | 6.942E-05 | 0.006038 |
| *CAV1* | 1 | 3 | 1.948307 | 0.4689484 | 1 |
| *CAV2* | 0 | 1 | 0 | 1 | 1 |
| *CDC42BPA* | 2 | 10 | 1.169251 | 0.6913335 | 1 |
| *CDC7* | 2 | 8 | 1.461676 | 0.6471773 | 1 |
| *CDKN2A* | 3 | 4 | 4.379679 | 0.0698255 | 1 |
| *CDKN2B* | 0 | 0 | NA | 1 | 1 |
| *CHEK2* | 1 | 2 | 2.927807 | 0.3774745 | 1 |
| *CHSY1* | 0 | 4 | 0 | 1 | 1 |
| *COL4A3* | 2 | 33 | 0.354859 | 0.221211 | 1 |
| *COL5A1* | 3 | 10 | 1.752807 | 0.4216144 | 1 |
| *COL8A1* | 1 | 4 | 1.459447 | 0.5470427 | 1 |
| *COL8A2* | 2 | 8 | 1.24893 | 0.6771928 | 1 |
| *CWC27* | 5 | 3 | 6.893048 | 0.0094737 | 0.824212 |
| *DCAF4L2* | 1 | 5 | 1.170053 | 1 | 1 |
| *DDHD1* | 1 | 12 | 0.426025 | 0.7065254 | 1 |
| *DHRS3* | 1 | 7 | 0.835498 | 1 | 1 |
| *DUSP1* | 1 | 1 | 3.716578 | 0.3796932 | 1 |
| *EFEMP1* | 0 | 2 | 0 | 1 | 1 |
| *ELOVL5* | 6 | 3 | 3.240642 | 0.0942742 | 1 |
| *ELP4* | 4 | 28 | 0.836516 | 1 | 1 |
| *EXOC2* | 0 | 3 | 0 | 1 | 1 |
| *F5* | 3 | 13 | 1.350185 | 0.7187271 | 1 |
| *FAM101A* | 0 | 3 | 0 | 1 | 1 |
| *FAM46A* | 0 | 4 | 0 | 1 | 1 |
| *FGF9* | 1 | 0 | NA | 0.1461988 | 1 |
| *FLNB* | 6 | 40 | 0.876423 | 1 | 1 |
| *FNDC3B* | 0 | 3 | 0 | 1 | 1 |
| *FOXC1* | 1 | 1 | 3.195187 | 0.4205403 | 1 |
| *FOXO1* | 0 | 5 | 0 | 1 | 1 |
| *GLCCI1* | 0 | 3 | 0 | 1 | 1 |
| *GLT8D2* | 0 | 1 | 0 | 1 | 1 |
| *GMDS* | 0 | 2 | 0 | 1 | 1 |
| *GPR15* | 1 | 9 | 0.650624 | 1 | 1 |
| *HORMAD2* | 3 | 16 | 1.096257 | 0.750556 | 1 |
| *HS3ST3B1* | 3 | 1 | 4.235294 | 0.3134333 | 1 |
| *HSF2* | 1 | 2 | 2.927807 | 0.3774745 | 1 |
| *IBTK* | 0 | 5 | 0 | 1 | 1 |
| *ICA1* | 2 | 3 | 3.765894 | 0.1662715 | 1 |
| *KCNMB2* | 0 | 2 | 0 | 1 | 1 |
| *KPNB1* | 1 | 0 | NA | 0.1461988 | 1 |
| *LCN12* | 0 | 1 | 0 | 1 | 1 |
| *LPAR1* | 0 | 1 | 0 | 1 | 1 |
| *LRRK1* | 3 | 13 | 1.226381 | 0.7302581 | 1 |
| *MPDZ* | 15 | 83 | 1.058208 | 0.8839215 | 1 |
| *MVB12B* | 0 | 1 | 0 | 1 | 1 |
| *NFIB* | 2 | 3 | 3.329055 | 0.1973911 | 1 |
| *NUP160* | 1 | 11 | 0.532329 | 1 | 1 |
| *PLCE1* | 2 | 20 | 0.585495 | 0.7598727 | 1 |
| *PMM2* | 1 | 6 | 0.975936 | 1 | 1 |
| *PTPRJ* | 5 | 15 | 1.951515 | 0.1995138 | 1 |
| *RAPSN* | 2 | 9 | 1.258467 | 0.6752564 | 1 |
| *RARB* | 0 | 2 | 0 | 1 | 1 |
| *RERE* | 5 | 5 | 5.49052 | 0.0113591 | 0.988242 |
| *RPAP3* | 2 | 12 | 0.975267 | 1 | 1 |
| *RXRA* | 3 | 3 | 5.106952 | 0.0600931 | 1 |
| *SALL1* | 2 | 10 | 1.154724 | 0.6943531 | 1 |
| *SGCG* | 1 | 7 | 0.835943 | 1 | 1 |
| *SIX6* | 3 | 20 | 0.878075 | 1 | 1 |
| *SRBD1* | 6 | 27 | 1.300772 | 0.6173824 | 1 |
| *SSSCA1* | 0 | 1 | 0 | 1 | 1 |
| *TGFBR3* | 0 | 1 | 0 | 1 | 1 |
| *TIPARP* | 0 | 4 | 0 | 1 | 1 |
| *TJP1* | 0 | 19 | 0 | 0.0963562 | 1 |
| *TMCO1* | 0 | 2 | 0 | 1 | 1 |
| *TMTC2* | 0 | 3 | 0 | 1 | 1 |
| *TMTC2* | 0 | 3 | 0 | 1 | 1 |
| *TRIB2* | 0 | 3 | 0 | 1 | 1 |
| *TRIOBP* | 18 | 84 | 1.211585 | 0.4803774 | 1 |
| *TXNRD2* | 3 | 12 | 1.448243 | 0.4755275 | 1 |
| *USP37* | 5 | 4 | 7.319519 | 0.0051064 | 0.444257 |
| *VKORC1L1* | 1 | 4 | 1.460561 | 0.5467392 | 1 |
| *ZNF469* | 20 | 138 | 0.733251 | 0.2215127 | 1 |

**Supplementary Figure 1**: Chromatograms of all eight *CARD10* mutation carriers in the POAG cohort. Mutations in all carriers found on WES were successfully validated using direct sequencing. The sixth base from the left displays the mutation of interest.

Sample with chr22:37912044, c.G635A, p.R212H:

AG0168


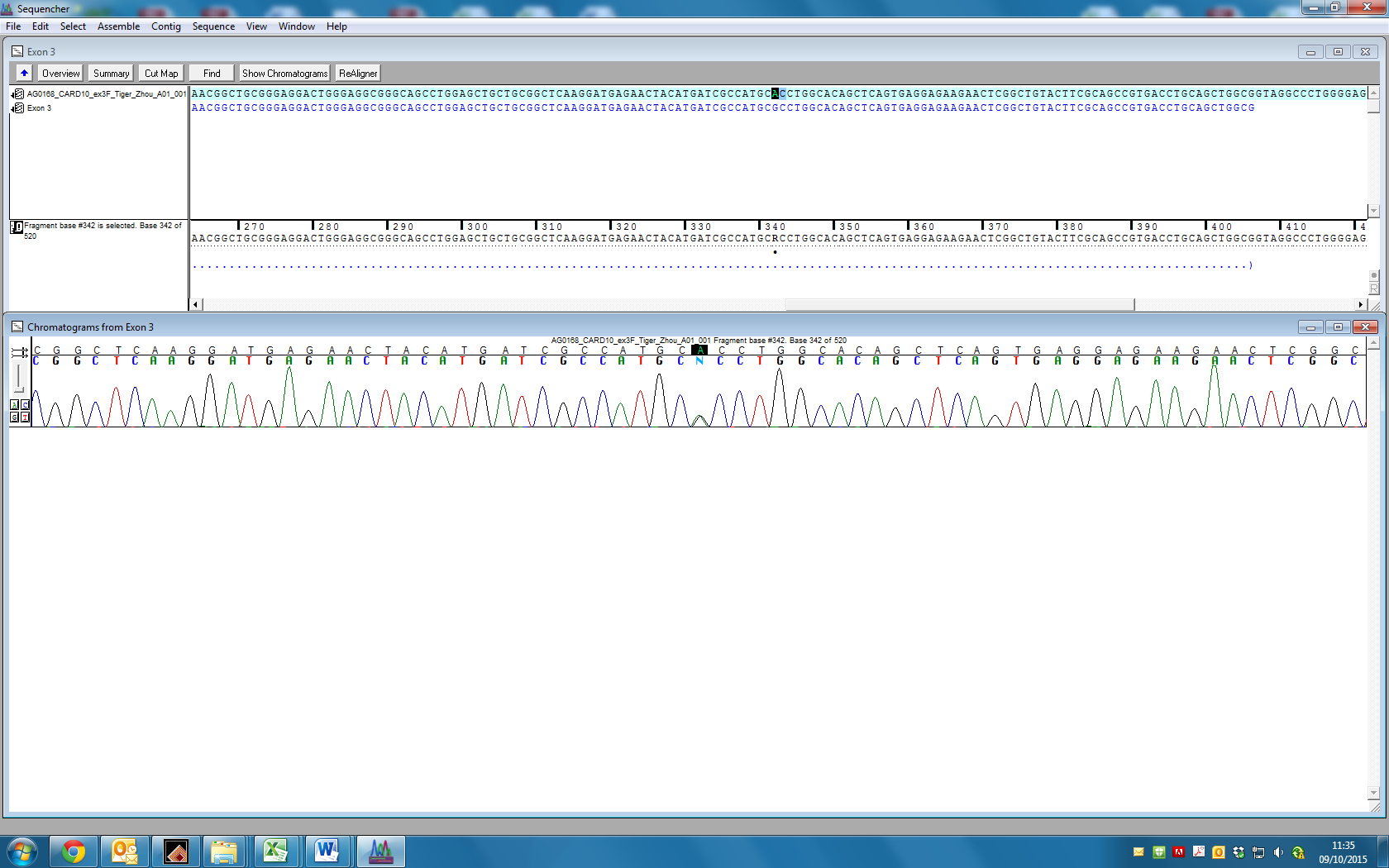


Samples with chr22:37904616, c.C983T, p.A328V:

AG1478

AG0679

AG0197


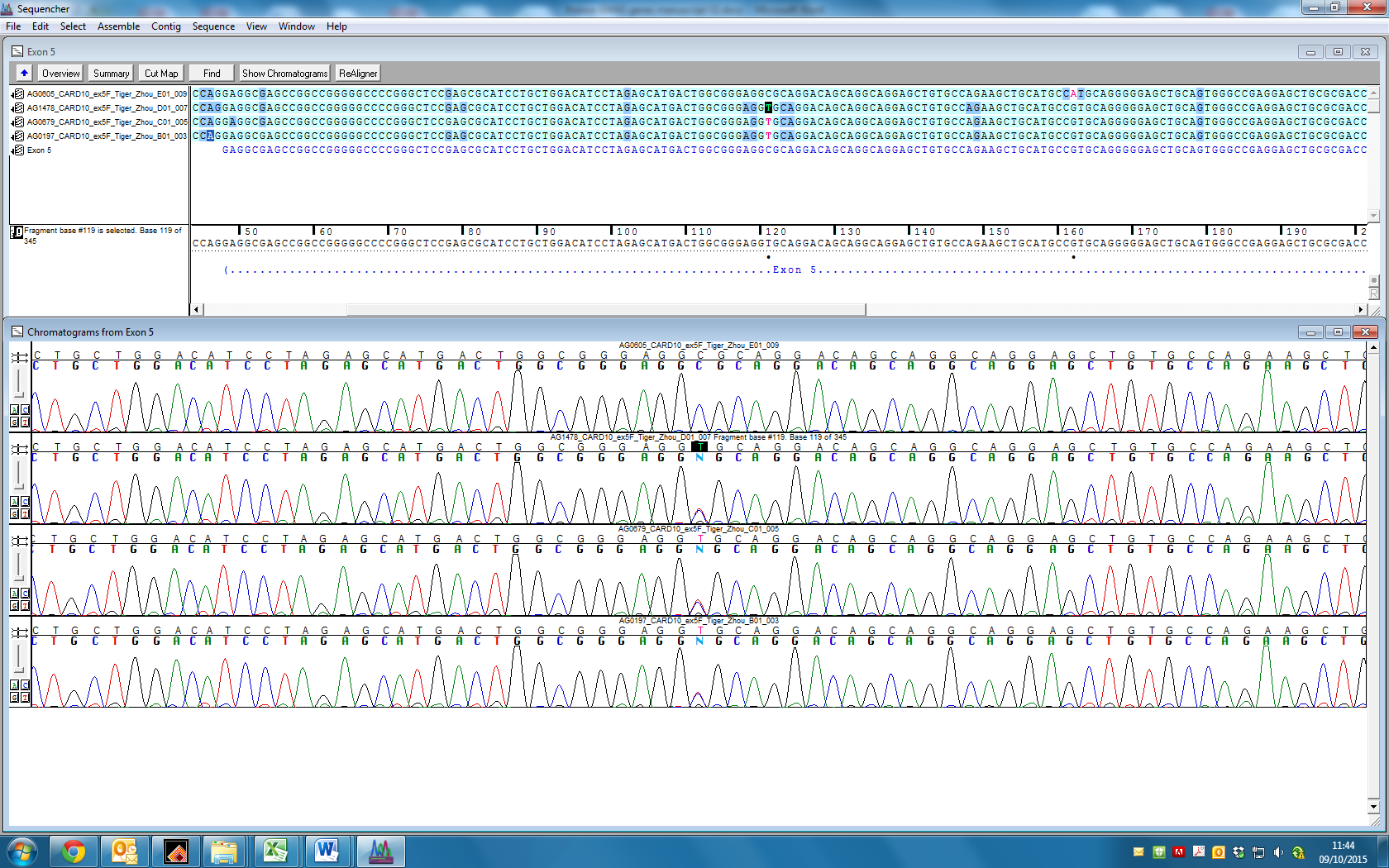

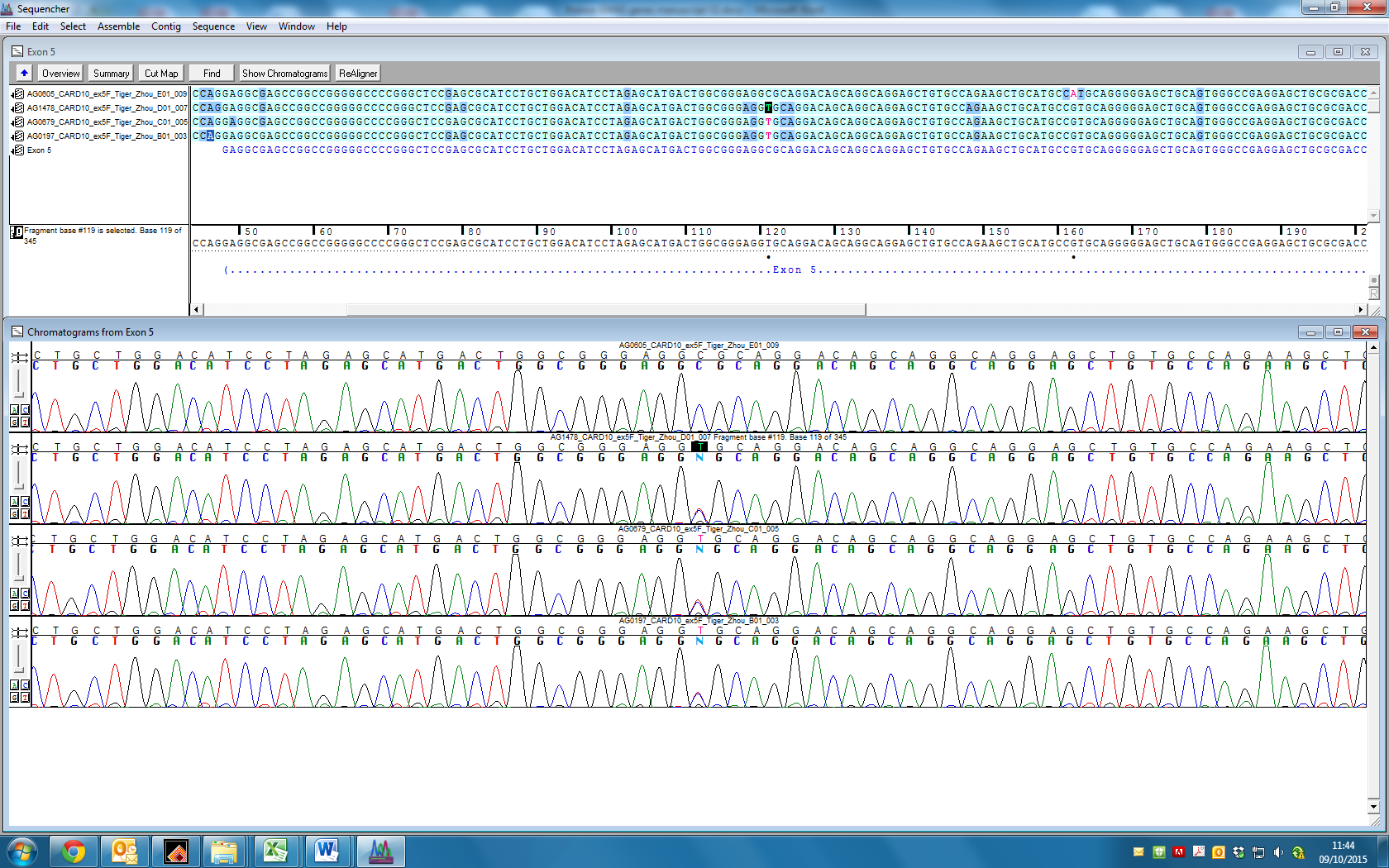

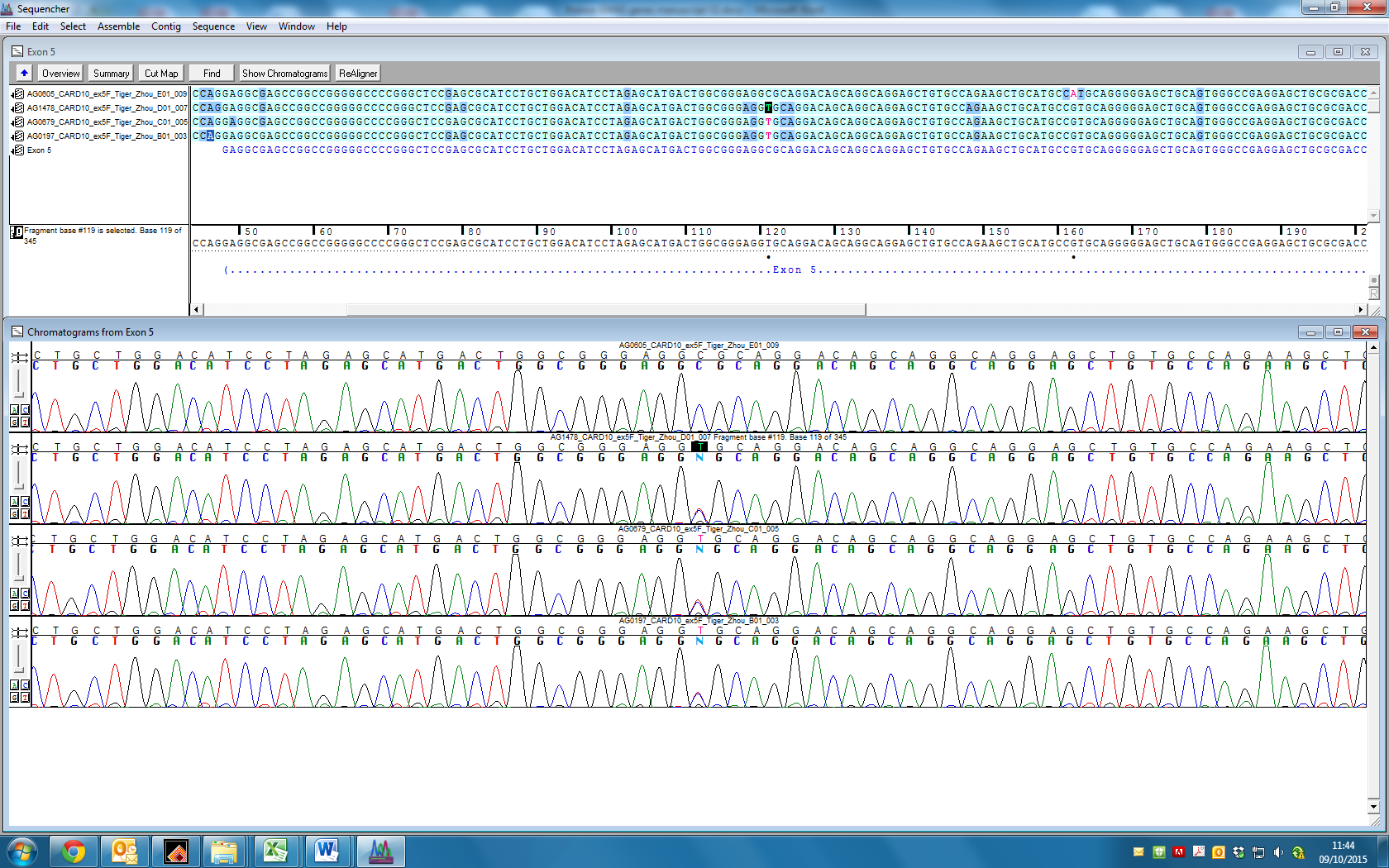


Sample with chr22:37904575, c.G1024A, p.V342M:

AG0605


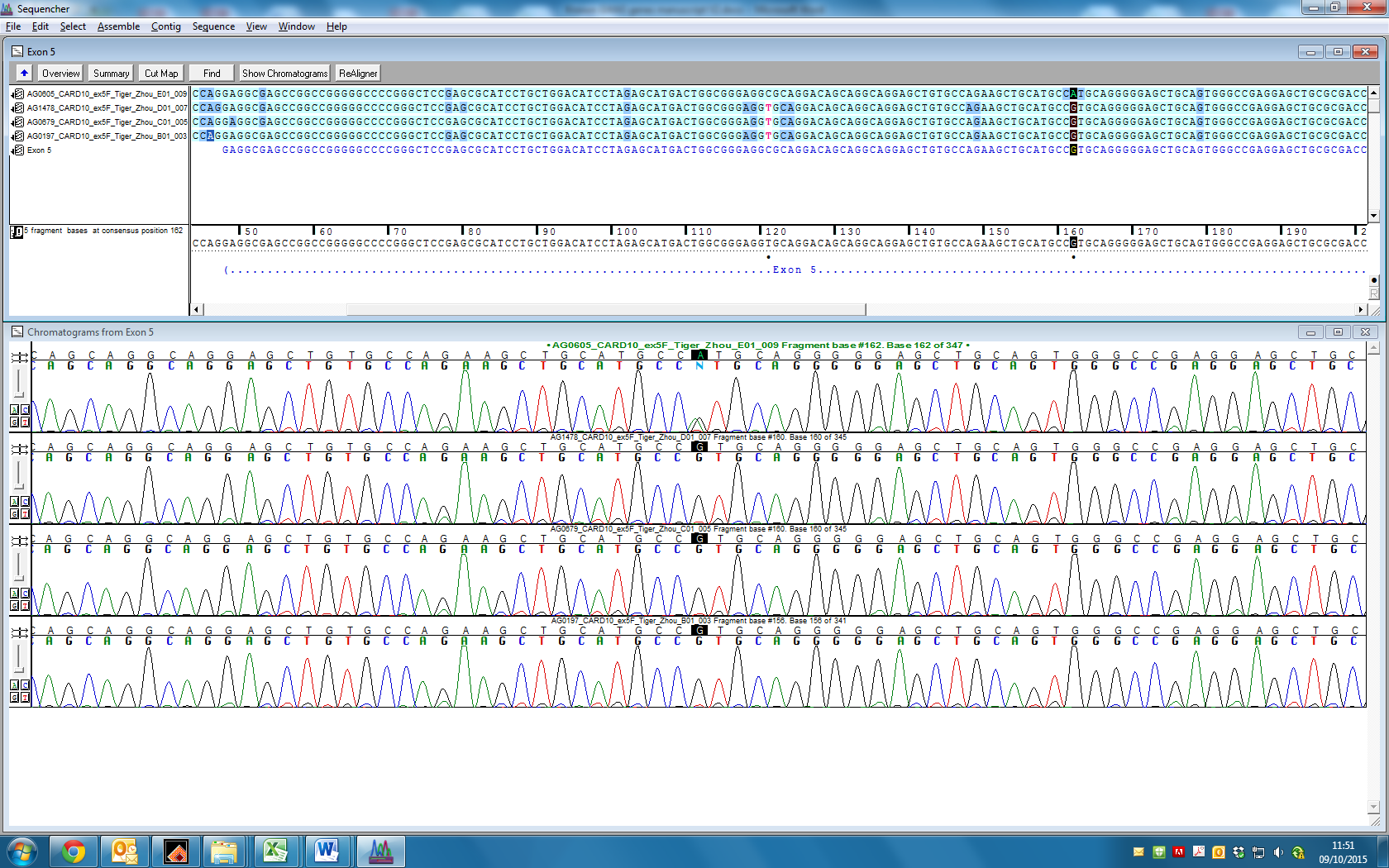


Sample with chr22:37902372, c.C1210T, p.R404W:

AG1733


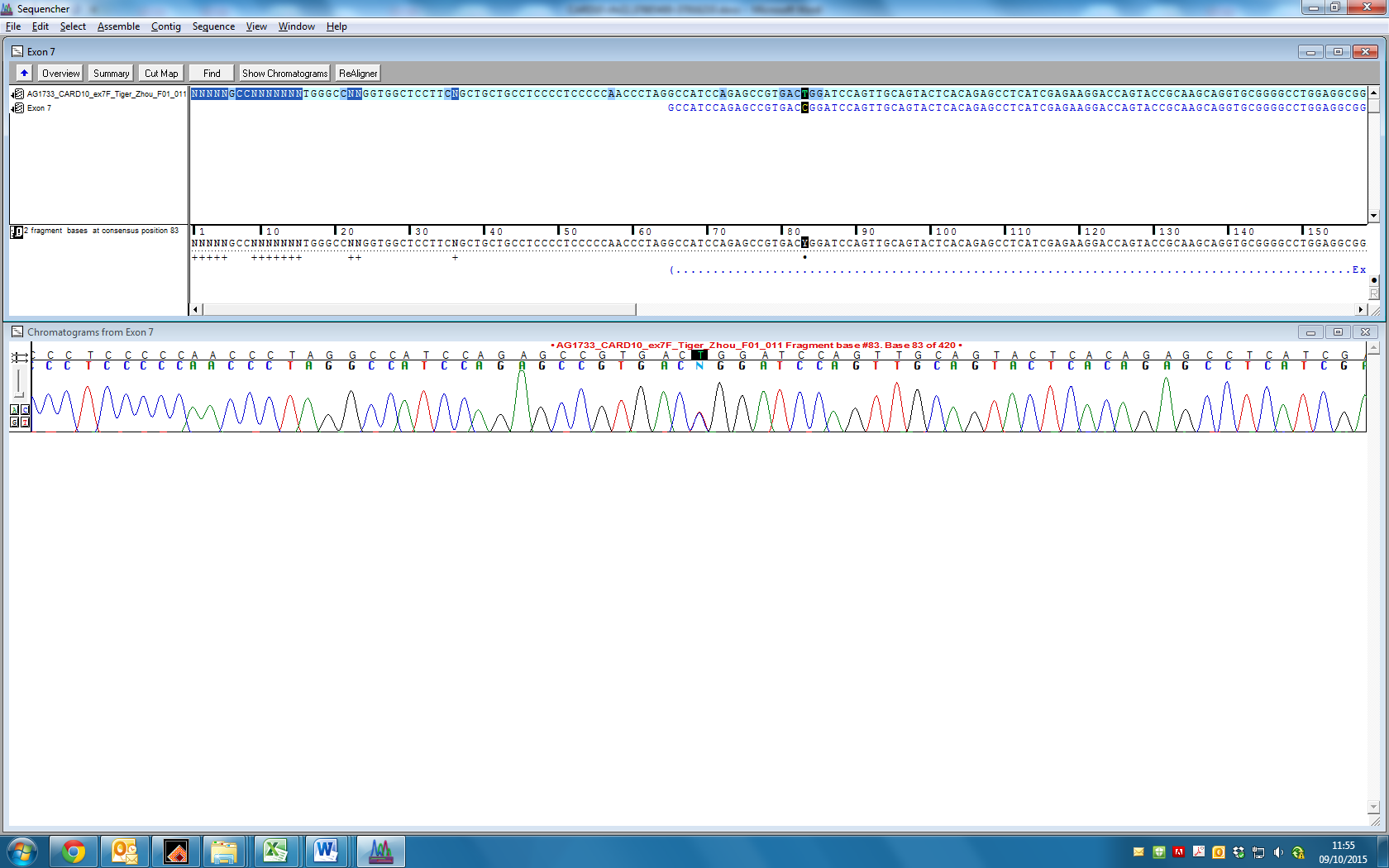


Samples with chr22:37888801, c.C2485T, p.R829W:

AG1751

AG1739


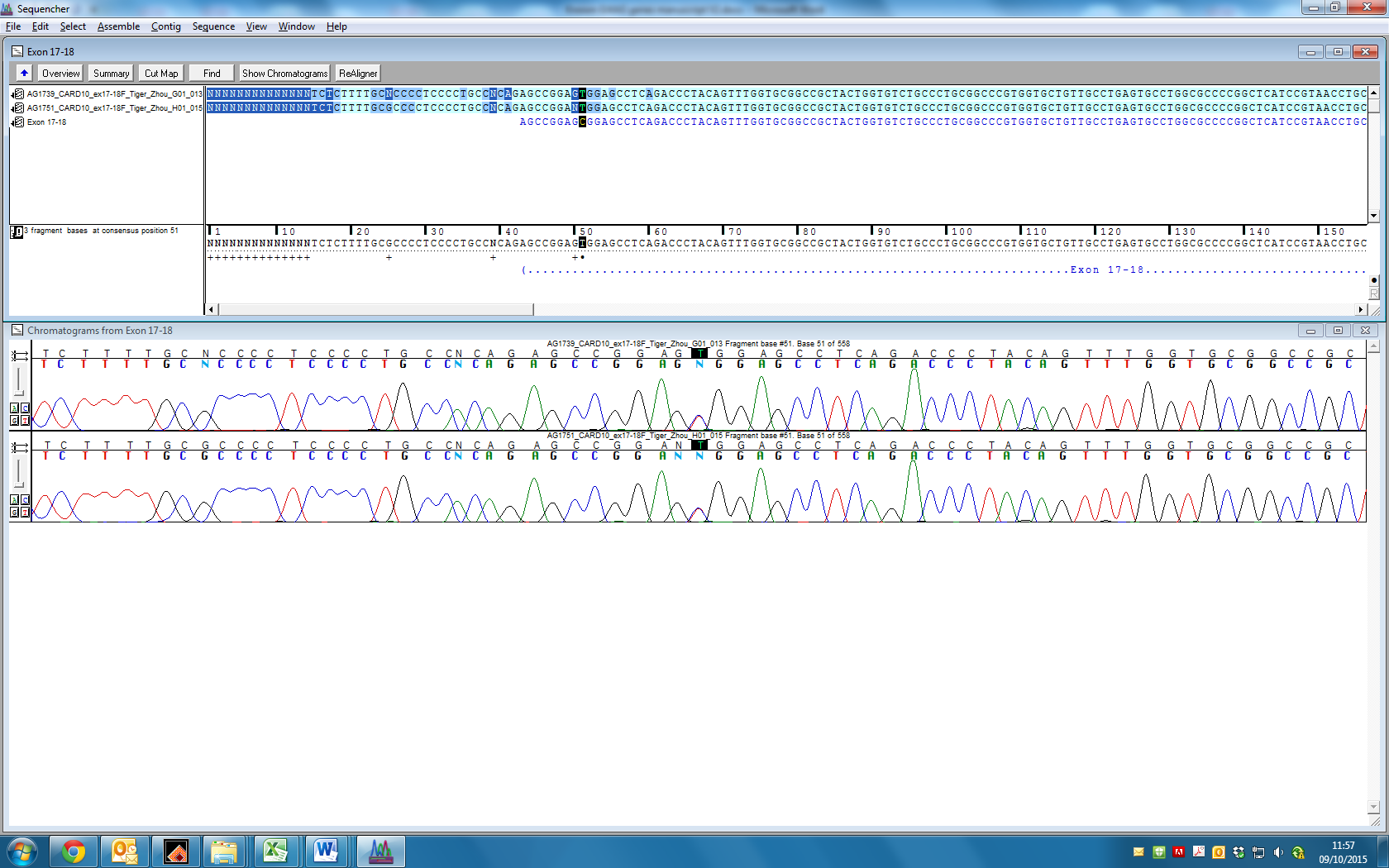

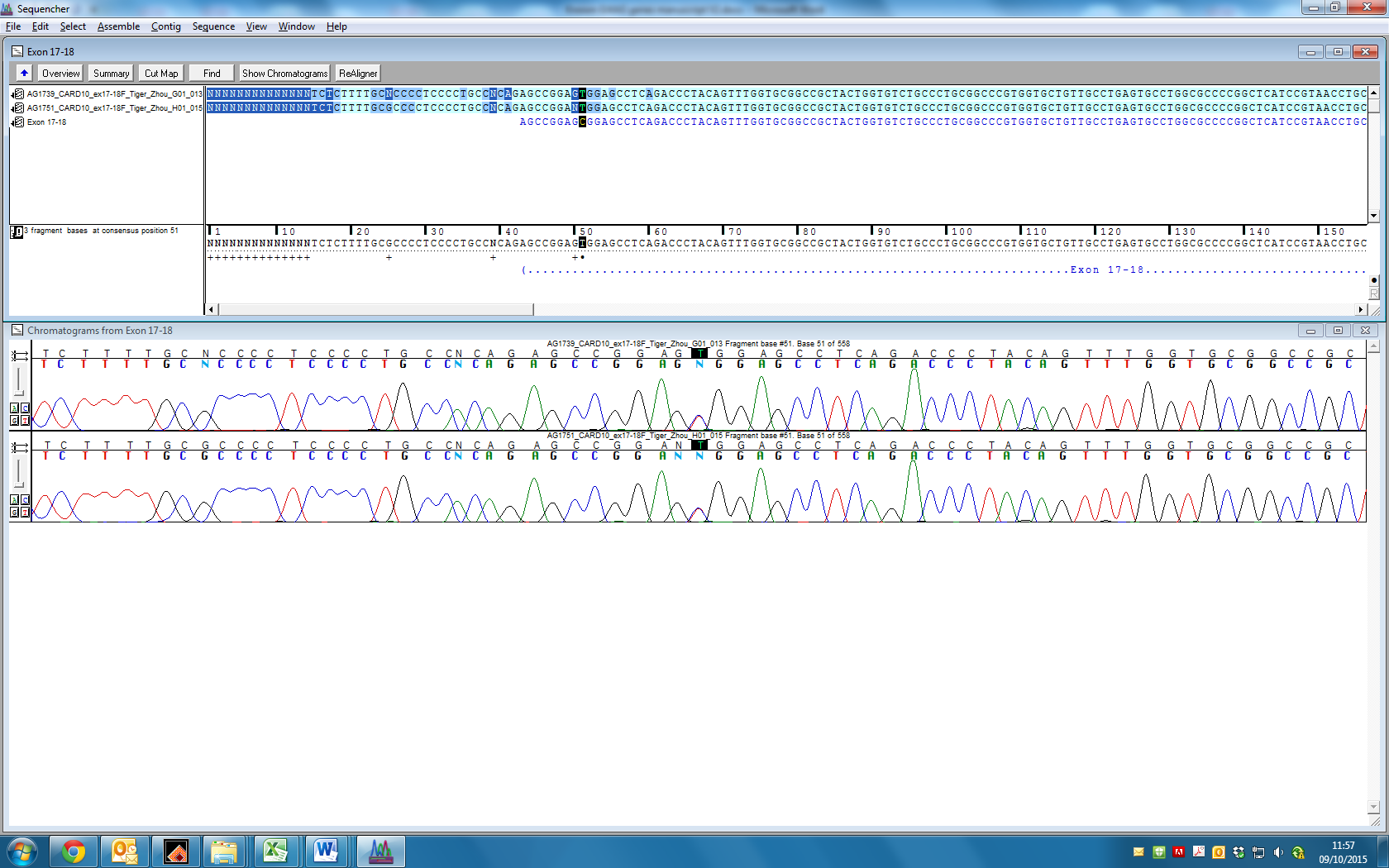


**Supplementary Table 4**: 86 randomly selected genes (Set 1 of 5).

| **Gene** | **POAG count** | **Control count** | **Odds ratio** | **Fisher's p value** | **Bonferroni corrected p** |
| --- | --- | --- | --- | --- | --- |
| *A1CF* | 2 | 2 | 5.85205 | 0.1048289 | 1 |
| *ADAMTS13* | 7 | 35 | 1.124465 | 0.8272367 | 1 |
| *AHCTF1* | 4 | 4 | 5.826453 | 0.0200615 | 1 |
| *ANKMY1* | 12 | 40 | 1.742435 | 0.1118611 | 1 |
| *ANO8* | 0 | 6 | 0 | 0.6013381 | 1 |
| *ARL14* | 1 | 2 | 2.927807 | 0.3774745 | 1 |
| *ATAD5* | 4 | 15 | 1.561477 | 0.5084975 | 1 |
| *ATR* | 1 | 6 | 0.970945 | 1 | 1 |
| *BEST3* | 2 | 4 | 2.927139 | 0.2147428 | 1 |
| *BTNL9* | 1 | 1 | 3.240642 | 0.4166409 | 1 |
| *C16orf58* | 1 | 11 | 0.53305 | 1 | 1 |
| *C1orf112* | 1 | 4 | 1.462121 | 0.546436 | 1 |
| *CADPS2* | 3 | 35 | 0.501416 | 0.3518734 | 1 |
| *CBR3* | 1 | 2 | 2.927807 | 0.3774745 | 1 |
| *CCDC18* | 2 | 23 | 0.507944 | 0.5666427 | 1 |
| *CCKBR* | 1 | 11 | 0.510938 | 1 | 1 |
| *CCL5* | 2 | 6 | 1.951872 | 0.3307526 | 1 |
| *CEMP1* | 3 | 5 | 3.192513 | 0.1203588 | 1 |
| *COG3* | 1 | 0 | NA | 1 | 1 |
| *DAB1* | 1 | 0 | NA | 0.1463129 | 1 |
| *EFCC1* | 6 | 4 | 3.63 | 0.0730326 | 1 |
| *EHMT2* | 1 | 5 | 1.147594 | 1 | 1 |
| *ENAM* | 2 | 9 | 1.301099 | 0.6683562 | 1 |
| *ERP27* | 0 | 2 | 0 | 1 | 1 |
| *ERVV-2* | 0 | 2 | 0 | 0.2193723 | 1 |
| *FAM134A* | 0 | 5 | 0 | 1 | 1 |
| *FAM186B* | 3 | 16 | 1.097594 | 0.7503793 | 1 |
| *FBXO3* | 0 | 2 | 0 | 1 | 1 |
| *FDX1* | 0 | 1 | 0 | 1 | 1 |
| *FRAS1* | 16 | 104 | 0.900741 | 0.7916693 | 1 |
| *FUT10* | 2 | 25 | 0.468327 | 0.4136134 | 1 |
| *GBE1* | 2 | 17 | 0.688756 | 1 | 1 |
| *GSTA1* | 0 | 2 | 0 | 1 | 1 |
| *HFM1* | 3 | 7 | 2.503329 | 0.1710959 | 1 |
| *HHIP* | 2 | 4 | 2.926916 | 0.2148756 | 1 |
| *HRNR* | 1 | 3 | 1.950535 | 0.4685225 | 1 |
| *IFI30* | 1 | 4 | 1.158088 | 1 | 1 |
| *ITGAE* | 1 | 18 | 0.32518 | 0.3429425 | 1 |
| *KDELR1* | 0 | 1 | 0 | 1 | 1 |
| *KDM3B* | 2 | 5 | 2.342674 | 0.2728739 | 1 |
| *L3MBTL1* | 3 | 18 | 0.975579 | 1 | 1 |
| *LDLRAP1* | 0 | 2 | 0 | 1 | 1 |
| *LOC100505841* | 3 | 0 | NA | 0.2626683 | 1 |
| *LPO* | 2 | 16 | 0.730318 | 1 | 1 |
| *LRRC1* | 2 | 7 | 1.604889 | 0.6330924 | 1 |
| *MAPK9* | 0 | 1 | 0 | 1 | 1 |
| *METTL21A* | 0 | 2 | 0 | 1 | 1 |
| *MRPL19* | 1 | 3 | 1.950535 | 0.4685225 | 1 |
| *MSMB* | 0 | 3 | 0 | 1 | 1 |
| *MTMR10* | 2 | 7 | 1.598167 | 0.6336662 | 1 |
| *MX1* | 6 | 21 | 1.665393 | 0.2722551 | 1 |
| *NIP7* | 0 | 2 | 0 | 1 | 1 |
| *NOP14* | 9 | 37 | 1.421323 | 0.3974909 | 1 |
| *NOTCH1* | 2 | 35 | 0.29892 | 0.1084715 | 1 |
| *NR2E1* | 1 | 0 | NA | 0.1461988 | 1 |
| *OPLAH* | 4 | 60 | 0.348289 | 0.0354409 | 1 |
| *OPN3* | 2 | 26 | 0.449404 | 0.4163838 | 1 |
| *OR10A5* | 0 | 5 | 0 | 1 | 1 |
| *OR6C4* | 3 | 8 | 2.195856 | 0.2100524 | 1 |
| *OVGP1* | 4 | 13 | 1.797844 | 0.2980062 | 1 |
| *PAQR4* | 0 | 9 | 0 | 0.3701724 | 1 |
| *PARP12* | 0 | 1 | 0 | 1 | 1 |
| *PARP8* | 1 | 4 | 1.464349 | 0.5458305 | 1 |
| *PMPCB* | 3 | 8 | 2.164773 | 0.2154121 | 1 |
| *PRR14* | 0 | 3 | 0 | 1 | 1 |
| *RBFA* | 0 | 4 | 0 | 1 | 1 |
| *RNF26* | 0 | 4 | 0 | 1 | 1 |
| *RPS6KB2* | 6 | 14 | 2.413579 | 0.1066764 | 1 |
| *SCN8A* | 2 | 8 | 1.446357 | 0.6489218 | 1 |
| *SGCB* | 0 | 2 | 0 | 1 | 1 |
| *SGPL1* | 0 | 6 | 0 | 0.6017614 | 1 |
| *SLA2* | 1 | 6 | 0.971658 | 1 | 1 |
| *SLC15A5* | 2 | 1 | 1.786096 | 1 | 1 |
| *SLC34A2* | 5 | 22 | 1.323286 | 0.5821436 | 1 |
| *SYCE2* | 5 | 14 | 2.091291 | 0.1824342 | 1 |
| *TAF1D* | 0 | 2 | 0 | 1 | 1 |
| *TCF3* | 4 | 28 | 0.787406 | 0.8080159 | 1 |
| *TMEM198* | 2 | 16 | 0.715775 | 1 | 1 |
| *TRAF7* | 1 | 1 | 4.5 | 0.3311565 | 1 |
| *TST* | 2 | 1 | 11.66845 | 0.0583192 | 1 |
| *WDR31* | 1 | 5 | 1.171658 | 1 | 1 |
| *WDR91* | 0 | 6 | 0 | 0.6016694 | 1 |
| *ZCRB1* | 0 | 1 | 0 | 1 | 1 |
| *ZNF296* | 0 | 2 | 0 | 1 | 1 |
| *ZNF649* | 0 | 7 | 0 | 0.6029747 | 1 |
| *ZPBP2* | 6 | 16 | 2.195053 | 0.1214391 | 1 |

**Supplementary Table 5**: 86 randomly selected genes (Set 2 of 5).

| **Gene** | **POAG count** | **Control count** | **Odds ratio** | **Fisher's p value** | **Bonferroni corrected p** |
| --- | --- | --- | --- | --- | --- |
| *AATK* | 3 | 3 | 3.88057 | 0.1059226 | 1 |
| *ACOT4* | 3 | 4 | 4.393048 | 0.069347 | 1 |
| *ADCK2* | 6 | 21 | 1.66794 | 0.2718428 | 1 |
| *ADTRP* | 1 | 1 | 5.855615 | 0.2709749 | 1 |
| *ALPPL2* | 2 | 4 | 2.901515 | 0.2174225 | 1 |
| *AOC2* | 1 | 8 | 0.727189 | 1 | 1 |
| *APOL1* | 5 | 12 | 2.43984 | 0.0911279 | 1 |
| *ARHGAP25* | 6 | 17 | 2.066688 | 0.1345317 | 1 |
| *ATAT1* | 0 | 1 | 0 | 1 | 1 |
| *ATRNL1* | 1 | 13 | 0.391032 | 0.4896112 | 1 |
| *BMP5* | 1 | 2 | 2.926916 | 0.3775991 | 1 |
| *BNIPL* | 2 | 3 | 3.900178 | 0.1581839 | 1 |
| *BRD1* | 1 | 3 | 1.942246 | 0.4699452 | 1 |
| *C15orf48* | 1 | 1 | 5.850267 | 0.2711696 | 1 |
| *C16orf96* | 4 | 27 | 0.852106 | 1 | 1 |
| *C4orf26* | 0 | 3 | 0 | 1 | 1 |
| *C9orf89* | 2 | 4 | 2.876114 | 0.2200136 | 1 |
| *CABIN1* | 7 | 51 | 0.791505 | 0.7082924 | 1 |
| *CCDC113* | 1 | 2 | 2.927807 | 0.3774745 | 1 |
| *CCDC159* | 0 | 7 | 0 | 0.6029715 | 1 |
| *CCIN* | 1 | 14 | 0.418204 | 0.7113386 | 1 |
| *CCNL1* | 2 | 2 | 5.855615 | 0.104756 | 1 |
| *CDCA8* | 0 | 5 | 0 | 1 | 1 |
| *CDK20* | 2 | 4 | 2.927807 | 0.2147428 | 1 |
| *CES4A* | 0 | 5 | 0 | 1 | 1 |
| *CFAP61* | 2 | 18 | 0.64099 | 0.7563381 | 1 |
| *CHL1* | 2 | 24 | 0.487857 | 0.5718871 | 1 |
| *CHP1* | 1 | 0 | NA | 0.1504212 | 1 |
| *COLEC12* | 0 | 2 | 0 | 1 | 1 |
| *DAZAP2* | 0 | 6 | 0 | 0.6017614 | 1 |
| *DEFB132* | 0 | 16 | 0 | 0.1504114 | 1 |
| *EHD3* | 1 | 4 | 1.463636 | 0.5459818 | 1 |
| *EPS15L1* | 1 | 0 | NA | 0.1461988 | 1 |
| *FAM117A* | 0 | 5 | 0 | 1 | 1 |
| *FAM193B* | 4 | 17 | 1.349733 | 0.539973 | 1 |
| *FAM196B* | 1 | 8 | 0.729412 | 1 | 1 |
| *FAXC* | 1 | 7 | 0.831169 | 1 | 1 |
| *FBXL14* | 3 | 16 | 1.044786 | 1 | 1 |
| *GTF2H1* | 0 | 1 | 0 | 1 | 1 |
| *HAT1* | 1 | 3 | 1.951872 | 0.4683807 | 1 |
| *HEATR5A* | 8 | 54 | 0.835446 | 0.8566907 | 1 |
| *HM13* | 0 | 1 | 0 | 1 | 1 |
| *HOXC8* | 0 | 1 | 0 | 1 | 1 |
| *IKBKE* | 3 | 18 | 0.973262 | 1 | 1 |
| *ING2* | 0 | 1 | 0 | 1 | 1 |
| *ITGBL1* | 1 | 12 | 0.487879 | 0.7063688 | 1 |
| *KARS* | 3 | 29 | 0.605854 | 0.6121912 | 1 |
| *KRT71* | 1 | 26 | 0.223673 | 0.1647471 | 1 |
| *LIG3* | 1 | 5 | 1.171123 | 1 | 1 |
| *NEK10* | 1 | 6 | 0.699049 | 1 | 1 |
| *NHP2* | 0 | 2 | 0 | 1 | 1 |
| *NIPSNAP3A* | 3 | 10 | 1.755615 | 0.4211953 | 1 |
| *NISCH* | 4 | 13 | 1.518268 | 0.5111687 | 1 |
| *NUCB2* | 1 | 0 | NA | 0.1467136 | 1 |
| *NUP88* | 0 | 6 | 0 | 0.6014656 | 1 |
| *OR10H2* | 2 | 4 | 2.927807 | 0.2147428 | 1 |
| *PDCD11* | 7 | 53 | 0.773336 | 0.7100059 | 1 |
| *PINX1* | 1 | 11 | 0.532268 | 1 | 1 |
| *PPP1R16A* | 2 | 9 | 1.003367 | 1 | 1 |
| *PRMT3* | 1 | 10 | 0.584492 | 1 | 1 |
| *PRR14* | 0 | 3 | 0 | 1 | 1 |
| *PRSS41* | 1 | 30 | 0.194831 | 0.0750453 | 1 |
| *RECQL4* | 16 | 89 | 0.966543 | 1 | 1 |
| *RHCE* | 0 | 1 | 0 | 1 | 1 |
| *RPL7A* | 3 | 17 | 1.033344 | 1 | 1 |
| *SORBS1* | 7 | 20 | 1.764643 | 0.1947405 | 1 |
| *SPHK2* | 1 | 5 | 1.039572 | 1 | 1 |
| *SPON2* | 3 | 2 | 7.692513 | 0.0338768 | 1 |
| *STK33* | 0 | 3 | 0 | 1 | 1 |
| *TARSL2* | 1 | 4 | 1.13135 | 1 | 1 |
| *TCAIM* | 0 | 7 | 0 | 0.602978 | 1 |
| *TCHH* | 0 | 20 | 0 | 0.0602135 | 1 |
| *TGFB2* | 2 | 7 | 1.647059 | 0.6297847 | 1 |
| *TRAF7* | 1 | 1 | 4.5 | 0.3311565 | 1 |
| *TRAPPC9* | 4 | 17 | 1.372968 | 0.535401 | 1 |
| *TRIM63* | 1 | 13 | 0.44626 | 0.7073792 | 1 |
| *TSPAN1* | 0 | 2 | 0 | 1 | 1 |
| *TXNRD3NB* | 1 | 5 | 1.168984 | 1 | 1 |
| *ZBTB8OS* | 1 | 3 | 1.950089 | 0.4686644 | 1 |
| *ZFR2* | 3 | 11 | 1.391763 | 0.7141015 | 1 |
| *ZNF286A* | 0 | 8 | 0 | 0.6121791 | 1 |
| *ZNF560* | 2 | 8 | 1.463755 | 0.646954 | 1 |
| *ZNF664* | 0 | 1 | 0 | 1 | 1 |
| *ZNF786* | 2 | 7 | 1.504966 | 0.6424142 | 1 |
| *ZNF829* | 0 | 2 | 0 | 1 | 1 |
| *ZNHIT3* | 0 | 3 | 0 | 1 | 1 |

**Supplementary Table 6**: 86 randomly selected genes (Set 3 of 5).

| **Gene** | **POAG count** | **Control count** | **Odds ratio** | **Fisher's p value** | **Bonferroni corrected p** |
| --- | --- | --- | --- | --- | --- |
| *AARSD1* | 0 | 3 | 0 | 1 | 1 |
| *ABCA1* | 2 | 19 | 0.61624 | 0.7573342 | 1 |
| *ACSL1* | 0 | 2 | 0 | 1 | 1 |
| *ADGRB3* | 2 | 11 | 1.064268 | 1 | 1 |
| *ADGRF2* | 7 | 36 | 1.136902 | 0.6680412 | 1 |
| *AHSG* | 1 | 25 | 0.233497 | 0.1618712 | 1 |
| *ARHGAP29* | 2 | 8 | 1.459626 | 0.6474015 | 1 |
| *ARPC4-TTLL3* | 1 | 12 | 0.485406 | 0.7062143 | 1 |
| *BRINP3* | 3 | 10 | 1.755258 | 0.4212998 | 1 |
| *C2* | 1 | 30 | 0.191711 | 0.0745187 | 1 |
| *C2CD4B* | 1 | 0 | NA | 1 | 1 |
| *CDH16* | 2 | 19 | 0.615649 | 0.7573527 | 1 |
| *CDH18* | 0 | 4 | 0 | 1 | 1 |
| *CFHR2* | 0 | 1 | 0 | 1 | 1 |
| *CLASP1* | 4 | 17 | 1.377233 | 0.5345755 | 1 |
| *CTNNA3* | 2 | 16 | 0.731746 | 1 | 1 |
| *CTNNBL1* | 0 | 2 | 0 | 1 | 1 |
| *DPYSL5* | 0 | 3 | 0 | 1 | 1 |
| *EARS2* | 0 | 5 | 0 | 1 | 1 |
| *EIF3B* | 3 | 9 | 1.951872 | 0.400524 | 1 |
| *FAM126A* | 3 | 16 | 1.096257 | 0.750556 | 1 |
| *FKBP3* | 1 | 2 | 2.77139 | 0.3926209 | 1 |
| *FLCN* | 2 | 7 | 1.668755 | 0.6282273 | 1 |
| *FSD1* | 1 | 1 | 5.762032 | 0.2744232 | 1 |
| *FZD6* | 3 | 4 | 4.392513 | 0.0694151 | 1 |
| *GALNT18* | 0 | 9 | 0 | 0.3734094 | 1 |
| *GDF6* | 1 | 2 | 2.894385 | 0.3806141 | 1 |
| *GJB7* | 0 | 9 | 0 | 0.3734094 | 1 |
| *GNB1L* | 1 | 6 | 0.863458 | 1 | 1 |
| *GPC5* | 0 | 12 | 0 | 0.2347457 | 1 |
| *GPR3* | 0 | 4 | 0 | 1 | 1 |
| *GSTP1* | 0 | 6 | 0 | 0.6017614 | 1 |
| *GUSB* | 0 | 8 | 0 | 0.6122945 | 1 |
| *GZMH* | 0 | 6 | 0 | 0.6017614 | 1 |
| *HINFP* | 3 | 17 | 1.033344 | 1 | 1 |
| *HMMR* | 0 | 3 | 0 | 1 | 1 |
| *IGSF5* | 1 | 9 | 0.650178 | 1 | 1 |
| *IL17RD* | 2 | 7 | 1.66864 | 0.6282273 | 1 |
| *KIF3B* | 0 | 1 | 0 | 1 | 1 |
| *KIT* | 0 | 3 | 0 | 1 | 1 |
| *KPRP* | 5 | 15 | 1.949279 | 0.1999143 | 1 |
| *KRTCAP2* | 0 | 6 | 0 | 0.6013985 | 1 |
| *LRRFIP2* | 4 | 7 | 3.346066 | 0.0645457 | 1 |
| *LUC7L* | 0 | 1 | 0 | 1 | 1 |
| *MAP4K2* | 4 | 28 | 0.832018 | 1 | 1 |
| *MAPK3* | 0 | 3 | 0 | 1 | 1 |
| *MED10* | 0 | 0 | NA | 1 | 1 |
| *MINOS1* | 1 | 1 | 3.160428 | 0.4235706 | 1 |
| *MORC2* | 0 | 7 | 0 | 0.6029747 | 1 |
| *MUS81* | 1 | 8 | 0.731618 | 1 | 1 |
| *N4BP1* | 0 | 1 | 0 | 1 | 1 |
| *NDRG3* | 1 | 1 | 5.855615 | 0.2709749 | 1 |
| *NDUFB6* | 1 | 11 | 0.506563 | 1 | 1 |
| *NEFH* | 1 | 7 | 0.752568 | 1 | 1 |
| *NEXN* | 2 | 13 | 0.89963 | 1 | 1 |
| *OR4Q3* | 0 | 8 | 0 | 0.6122366 | 1 |
| *OTUD3* | 1 | 23 | 0.181134 | 0.0669601 | 1 |
| *P4HA2* | 0 | 6 | 0 | 0.6017614 | 1 |
| *PDP2* | 0 | 1 | 0 | 1 | 1 |
| *PPAP2A* | 0 | 3 | 0 | 1 | 1 |
| *PRLHR* | 0 | 5 | 0 | 0.5900972 | 1 |
| *PTF1A* | 1 | 0 | NA | 0.1461988 | 1 |
| *PYGO2* | 1 | 2 | 2.925134 | 0.3777238 | 1 |
| *PZP* | 8 | 28 | 1.673033 | 0.2307263 | 1 |
| *RAD54L* | 6 | 29 | 1.210098 | 0.6311035 | 1 |
| *RHBDD1* | 2 | 7 | 1.673033 | 0.6279043 | 1 |
| *RYR3* | 18 | 79 | 1.332262 | 0.3056558 | 1 |
| *SCUBE1* | 3 | 16 | 1.085561 | 0.7525348 | 1 |
| *SLC34A3* | 3 | 9 | 1.394162 | 0.7114481 | 1 |
| *SLC41A1* | 0 | 2 | 0 | 1 | 1 |
| *SLC7A4* | 6 | 47 | 0.73708 | 0.6937788 | 1 |
| *SLFN12L* | 4 | 15 | 1.561497 | 0.508433 | 1 |
| *SMYD1* | 9 | 34 | 1.548196 | 0.2738271 | 1 |
| *SRL* | 1 | 2 | 2.910873 | 0.3791007 | 1 |
| *SYT10* | 1 | 3 | 1.951872 | 0.4683807 | 1 |
| *TALDO1* | 2 | 20 | 0.581952 | 0.7601671 | 1 |
| *TARBP2* | 1 | 8 | 0.727941 | 1 | 1 |
| *TAS2R16* | 0 | 1 | 0 | 1 | 1 |
| *TBC1D32* | 9 | 17 | 3.076096 | 0.0097235 | 0.836218 |
| *TBXA2R* | 3 | 24 | 0.665274 | 0.7892756 | 1 |
| *TCTN2* | 1 | 4 | 1.437166 | 0.5520959 | 1 |
| *TLCD2* | 1 | 25 | 0.233155 | 0.1618711 | 1 |
| *TOMM20L* | 0 | 1 | 0 | 1 | 1 |
| *TPRN* | 2 | 17 | 0.451068 | 0.3960126 | 1 |
| *TRIL* | 0 | 0 | NA | 1 | 1 |
| *UTP14C* | 4 | 15 | 1.561497 | 0.508433 | 1 |

**Supplementary Table 7**: 86 randomly selected genes (Set 4 of 5).

| **Gene** | **POAG count** | **Control count** | **Odds ratio** | **Fisher's p value** | **Bonferroni corrected p** |
| --- | --- | --- | --- | --- | --- |
| *ABCC9* | 0 | 11 | 0 | 0.3838643 | 1 |
| *ACOT6* | 0 | 2 | 0 | 1 | 1 |
| *ADD2* | 1 | 8 | 0.718416 | 1 | 1 |
| *ADORA3* | 1 | 3 | 1.793672 | 0.4948261 | 1 |
| *AFF1* | 8 | 26 | 1.791855 | 0.1464416 | 1 |
| *ANAPC16* | 1 | 4 | 1.463904 | 0.5459818 | 1 |
| *ANKRD46* | 1 | 0 | NA | 1 | 1 |
| *APOBEC3G* | 0 | 5 | 0 | 1 | 1 |
| *ARRDC5* | 0 | 5 | 0 | 1 | 1 |
| *ATP5SL* | 2 | 5 | 2.219608 | 0.2920291 | 1 |
| *ATP6V1D* | 3 | 6 | 2.927807 | 0.1331597 | 1 |
| *BMP3* | 2 | 11 | 1.015881 | 1 | 1 |
| *C21orf62* | 1 | 3 | 1.951872 | 0.4683807 | 1 |
| *C6orf25* | 1 | 1 | 5.631016 | 0.2793998 | 1 |
| *C7orf65* | 0 | 3 | 0 | 1 | 1 |
| *CAPRIN2* | 3 | 17 | 1.033134 | 1 | 1 |
| *CBS* | 3 | 21 | 0.807257 | 1 | 1 |
| *CCDC168* | 38 | 314 | 0.708517 | 0.0602607 | 1 |
| *CCDC183* | 0 | 4 | 0 | 1 | 1 |
| *CD200R1L* | 3 | 5 | 3.508556 | 0.0995173 | 1 |
| *CD27* | 1 | 4 | 1.443405 | 0.5507093 | 1 |
| *CFHR1* | 0 | 4 | 0 | 1 | 1 |
| *CLN6* | 0 | 2 | 0 | 1 | 1 |
| *COBL* | 3 | 19 | 0.856389 | 1 | 1 |
| *CRYZ* | 2 | 11 | 1.063685 | 1 | 1 |
| *DAB2IP* | 3 | 22 | 0.78541 | 1 | 1 |
| *DES* | 1 | 2 | 2.609626 | 0.4095845 | 1 |
| *DHRS1* | 3 | 4 | 4.392284 | 0.0694151 | 1 |
| *DIEXF* | 2 | 12 | 0.975045 | 1 | 1 |
| *DLGAP4* | 1 | 12 | 0.463012 | 0.7053515 | 1 |
| *DRAXIN* | 0 | 2 | 0 | 1 | 1 |
| *ELF3* | 1 | 4 | 1.461898 | 0.546436 | 1 |
| *ENDOD1* | 0 | 2 | 0 | 1 | 1 |
| *FAM189B* | 4 | 27 | 0.835443 | 1 | 1 |
| *FLG2* | 2 | 17 | 0.688162 | 1 | 1 |
| *GFI1* | 0 | 21 | 0 | 0.0604393 | 1 |
| *GNB1L* | 1 | 6 | 0.863458 | 1 | 1 |
| *GPR21* | 0 | 1 | 0 | 1 | 1 |
| *HEATR6* | 1 | 39 | 0.150075 | 0.0230659 | 1 |
| *HIF3A* | 1 | 11 | 0.476746 | 0.7037453 | 1 |
| *HIST1H4C* | 1 | 0 | NA | 0.1461419 | 1 |
| *IKZF2* | 3 | 4 | 4.391711 | 0.0694151 | 1 |
| *KCTD18* | 1 | 9 | 0.650386 | 1 | 1 |
| *KIF22* | 0 | 2 | 0 | 1 | 1 |
| *LCN10* | 0 | 3 | 0 | 1 | 1 |
| *LINGO2* | 0 | 1 | 0 | 1 | 1 |
| *MIDN* | 1 | 1 | 3.187166 | 0.4212358 | 1 |
| *MMP11* | 0 | 13 | 0 | 0.2379489 | 1 |
| *MROH9* | 1 | 5 | 1.170053 | 1 | 1 |
| *MSRB2* | 2 | 2 | 5.855615 | 0.104756 | 1 |
| *N4BP2L1* | 0 | 2 | 0 | 1 | 1 |
| *NAT14* | 1 | 1 | 4.566845 | 0.3275764 | 1 |
| *NINJ1* | 0 | 3 | 0 | 1 | 1 |
| *OR4S1* | 0 | 4 | 0 | 1 | 1 |
| *OR5D18* | 2 | 9 | 1.301446 | 0.6683562 | 1 |
| *PDE9A* | 2 | 4 | 2.927807 | 0.2147428 | 1 |
| *PGK2* | 2 | 9 | 1.301464 | 0.6683562 | 1 |
| *PIGM* | 2 | 7 | 1.67085 | 0.6280654 | 1 |
| *PLAC9* | 1 | 2 | 2.914439 | 0.3787242 | 1 |
| *PLXNA2* | 8 | 44 | 1.059796 | 0.8433038 | 1 |
| *PRKACG* | 0 | 4 | 0 | 1 | 1 |
| *PTH2R* | 1 | 8 | 0.731952 | 1 | 1 |
| *RARB* | 0 | 2 | 0 | 1 | 1 |
| *REM2* | 2 | 0 | NA | 0.0214227 | 1 |
| *RETNLB* | 0 | 2 | 0 | 1 | 1 |
| *RHOU* | 0 | 3 | 0 | 1 | 1 |
| *SDR42E1* | 0 | 8 | 0 | 0.6122366 | 1 |
| *SERPINI2* | 1 | 5 | 1.170053 | 1 | 1 |
| *SGK494* | 3 | 14 | 1.25234 | 0.7279438 | 1 |
| *SIGLEC15* | 0 | 1 | 0 | 1 | 1 |
| *SIT1* | 0 | 4 | 0 | 1 | 1 |
| *SLC13A4* | 1 | 2 | 2.890374 | 0.3809943 | 1 |
| *SLC5A7* | 1 | 20 | 0.292914 | 0.3467883 | 1 |
| *SLFNL1* | 6 | 22 | 1.504063 | 0.4250656 | 1 |
| *SNX33* | 2 | 8 | 1.460784 | 0.6473266 | 1 |
| *TARS2* | 1 | 11 | 0.531704 | 1 | 1 |
| *TCEB3B* | 0 | 4 | 0 | 1 | 1 |
| *TDRD5* | 5 | 25 | 1.170053 | 0.7934176 | 1 |
| *TRPA1* | 6 | 28 | 1.254393 | 0.6234452 | 1 |
| *TSKU* | 0 | 8 | 0 | 0.6151802 | 1 |
| *TTC39C* | 0 | 2 | 0 | 1 | 1 |
| *WDR83* | 1 | 5 | 1.158075 | 1 | 1 |
| *XPC* | 3 | 10 | 1.635963 | 0.4390256 | 1 |
| *ZC3H12A* | 0 | 21 | 0 | 0.0604527 | 1 |
| *ZFYVE16* | 1 | 6 | 0.975045 | 1 | 1 |
| *ZNF836* | 1 | 6 | 0.975936 | 1 | 1 |

**Supplementary Table 8**: 86 randomly selected genes (Set 5 of 5).

| **Gene** | **POAG count** | **Control count** | **Odds ratio** | **Fisher's p value** | **Bonferroni corrected p** |
| --- | --- | --- | --- | --- | --- |
| *ABCC1* | 3 | 24 | 0.731618 | 0.787467 | 1 |
| *ABCC5* | 0 | 6 | 0 | 0.6017614 | 1 |
| *ADAMTS15* | 1 | 9 | 0.635175 | 1 | 1 |
| *ALDH18A1* | 1 | 3 | 1.951426 | 0.4683807 | 1 |
| *ARHGAP9* | 6 | 23 | 1.441758 | 0.4347851 | 1 |
| *ARL6IP6* | 0 | 1 | 0 | 1 | 1 |
| *ASB3* | 3 | 1 | 2.679144 | 0.6265628 | 1 |
| *BAGE2* | 1 | 1 | 1.390374 | 1 | 1 |
| *BIVM-ERCC5* | 2 | 12 | 0.975681 | 1 | 1 |
| *C1orf115* | 1 | 18 | 0.321351 | 0.3421671 | 1 |
| *C1orf159* | 3 | 10 | 1.683957 | 0.4314019 | 1 |
| *C2CD3* | 5 | 17 | 1.646579 | 0.3645786 | 1 |
| *C6* | 6 | 32 | 1.097928 | 0.8166957 | 1 |
| *CACNA2D2* | 6 | 9 | 3.744652 | 0.0179085 | 1 |
| *CAMSAP3* | 1 | 7 | 0.745034 | 1 | 1 |
| *CCDC173* | 2 | 6 | 1.951872 | 0.3307526 | 1 |
| *CDK4* | 1 | 4 | 1.464238 | 0.5458305 | 1 |
| *CHRNA2* | 1 | 0 | NA | 0.1461988 | 1 |
| *COL27A1* | 6 | 26 | 1.328906 | 0.4607456 | 1 |
| *COL6A5* | 7 | 67 | 0.611723 | 0.2443287 | 1 |
| *DHX37* | 1 | 5 | 1.154439 | 1 | 1 |
| *EEF2* | 2 | 7 | 1.640183 | 0.630303 | 1 |
| *ENDOU* | 2 | 2 | 4.142602 | 0.1731901 | 1 |
| *EPHB2* | 4 | 13 | 1.799484 | 0.2977954 | 1 |
| *ERICH3* | 1 | 15 | 0.389967 | 0.4941027 | 1 |
| *FAM107A* | 0 | 8 | 0 | 0.6135303 | 1 |
| *FAM3B* | 1 | 7 | 0.777438 | 1 | 1 |
| *FBXL13* | 1 | 16 | 0.365642 | 0.4945189 | 1 |
| *FBXL6* | 0 | 4 | 0 | 1 | 1 |
| *FBXO24* | 2 | 8 | 1.394003 | 0.655376 | 1 |
| *FPGT* | 4 | 9 | 2.594573 | 0.1110443 | 1 |
| *FRY* | 4 | 10 | 2.342246 | 0.1371047 | 1 |
| *GABBR1* | 1 | 6 | 0.962567 | 1 | 1 |
| *GPSM3* | 0 | 3 | 0 | 1 | 1 |
| *H2AFZ* | 1 | 1 | 5.839572 | 0.2715599 | 1 |
| *HSPA4* | 0 | 3 | 0 | 1 | 1 |
| *IFNAR2* | 1 | 11 | 0.532329 | 1 | 1 |
| *IL17A* | 2 | 9 | 1.296257 | 0.6690938 | 1 |
| *INVS* | 2 | 15 | 0.780749 | 1 | 1 |
| *ITGB3BP* | 0 | 5 | 0 | 1 | 1 |
| *KBTBD13* | 3 | 4 | 1.707219 | 0.443393 | 1 |
| *KRTAP26-1* | 0 | 2 | 0 | 1 | 1 |
| *KRTAP5-9* | 1 | 11 | 0.529169 | 1 | 1 |
| *LAMA2* | 13 | 83 | 0.908738 | 0.8832301 | 1 |
| *LAPTM4B* | 1 | 3 | 1.3041 | 1 | 1 |
| *LIPJ* | 1 | 6 | 0.974153 | 1 | 1 |
| *LOC81691* | 0 | 7 | 0 | 0.6029747 | 1 |
| *LRCOL1* | 2 | 1 | 1.574332 | 1 | 1 |
| *MEIS2* | 1 | 3 | 1.950089 | 0.4686644 | 1 |
| *MTA1* | 1 | 1 | 3.468806 | 0.3981581 | 1 |
| *MYBPC2* | 2 | 18 | 0.632997 | 0.7562212 | 1 |
| *NOL12* | 1 | 0 | NA | 0.1511487 | 1 |
| *NUB1* | 2 | 3 | 3.881759 | 0.1592283 | 1 |
| *OR1C1* | 1 | 2 | 2.925134 | 0.3777238 | 1 |
| *OR1S1* | 2 | 11 | 1.064657 | 1 | 1 |
| *OTX2* | 1 | 0 | NA | 0.1463129 | 1 |
| *PFKFB4* | 0 | 3 | 0 | 1 | 1 |
| *PLA2G10* | 0 | 1 | 0 | 1 | 1 |
| *POLR2I* | 0 | 1 | 0 | 1 | 1 |
| *RCBTB2* | 0 | 5 | 0 | 1 | 1 |
| *RHOB* | 0 | 10 | 0 | 0.3769304 | 1 |
| *RPAIN* | 0 | 3 | 0 | 1 | 1 |
| *RSG1* | 1 | 6 | 0.962567 | 1 | 1 |
| *RTCA* | 0 | 2 | 0 | 1 | 1 |
| *SAP30L* | 1 | 1 | 5.855615 | 0.2709749 | 1 |
| *SCN11A* | 2 | 10 | 1.170945 | 0.6910151 | 1 |
| *SCN7A* | 14 | 60 | 1.363815 | 0.3167056 | 1 |
| *SERPINI2* | 1 | 5 | 1.170053 | 1 | 1 |
| *SETMAR* | 3 | 6 | 2.917494 | 0.1341161 | 1 |
| *SIGLEC6* | 1 | 5 | 1.168128 | 1 | 1 |
| *SIN3B* | 1 | 2 | 2.818182 | 0.3879669 | 1 |
| *SIX4* | 1 | 5 | 1.117005 | 1 | 1 |
| *SLC25A23* | 1 | 8 | 0.729278 | 1 | 1 |
| *SMYD4* | 1 | 13 | 0.450144 | 0.7072659 | 1 |
| *SULT4A1* | 0 | 1 | 0 | 1 | 1 |
| *SYCN* | 1 | 1 | 3.86631 | 0.3693644 | 1 |
| *TBC1D2* | 4 | 30 | 0.756726 | 0.8089525 | 1 |
| *TEP1* | 11 | 74 | 0.86684 | 0.756227 | 1 |
| *TTC37* | 3 | 9 | 1.951223 | 0.4005981 | 1 |
| *TTC39C* | 0 | 2 | 0 | 1 | 1 |
| *UGT2A1* | 2 | 6 | 1.897802 | 0.3420271 | 1 |
| *VPS33B* | 0 | 1 | 0 | 1 | 1 |
| *YWHAQ* | 0 | 3 | 0 | 1 | 1 |
| *ZBTB8B* | 0 | 6 | 0 | 0.6017153 | 1 |
| *ZFYVE19* | 2 | 24 | 0.487701 | 0.5719418 | 1 |
| *ZNF846* | 2 | 27 | 0.433749 | 0.4214984 | 1 |

**References**

Axenovich, T, Zorkoltseva, I, Belonogova, N, van Koolwijk, LM, Borodin, P, Kirichenko, A, Babenko, V, Ramdas, WD, Amin, N, Despriet, DD, Vingerling, JR, Lemij, HG et al. 2011. Linkage and association analyses of glaucoma related traits in a large pedigree from a Dutch genetically isolated population. J Med Genet 48**:** 802-9.

Bailey, JN, Loomis, SJ, Kang, JH, Allingham, RR, Gharahkhani, P, Khor, CC, Burdon, KP, Aschard, H, Chasman, DI, Igo, RP, Jr., Hysi, PG, Glastonbury, CA et al. 2016. Genome-wide association analysis identifies TXNRD2, ATXN2 and FOXC1 as susceptibility loci for primary open-angle glaucoma. Nat Genet 48**:** 189-94.

Burdon, KP, Macgregor, S, Hewitt, AW, Sharma, S, Chidlow, G, Mills, RA, Danoy, P, Casson, R, Viswanathan, AC, Liu, JZ, Landers, J, Henders, AK et al. 2011. Genome-wide association study identifies susceptibility loci for open angle glaucoma at TMCO1 and CDKN2B-AS1. Nat Genet 43**:** 574-8.

Chen, Y, Lin, Y, Vithana, EN, Jia, L, Zuo, X, Wong, TY, Chen, LJ, Zhu, X, Tam, PO, Gong, B, Qian, S, Li, Z et al. 2014. Common variants near ABCA1 and in PMM2 are associated with primary open-angle glaucoma. Nat Genet 46**:** 1115-9.

Gao, X, Gauderman, WJ, Liu, Y, Marjoram, P, Torres, M, Haritunians, T, Kuo, JZ, Chen, YD, Allingham, RR, Hauser, MA, Taylor, KD, Rotter, JI et al. 2013. A genome-wide association study of central corneal thickness in Latinos. Invest Ophthalmol Vis Sci 54**:** 2435-43.

Gharahkhani, P, Burdon, KP, Fogarty, R, Sharma, S, Hewitt, AW, Martin, S, Law, MH, Cremin, K, Bailey, JN, Loomis, SJ, Pasquale, LR, Haines, JL et al. 2014. Common variants near ABCA1, AFAP1 and GMDS confer risk of primary open-angle glaucoma. Nat Genet 46**:** 1120-5.

Hoehn, R, Zeller, T, Verhoeven, VJ, Grus, F, Adler, M, Wolfs, RC, Uitterlinden, AG, Castagne, R, Schillert, A, Klaver, CC, Pfeiffer, N , Mirshahi, A 2012. Population-based meta-analysis in Caucasians confirms association with COL5A1 and ZNF469 but not COL8A2 with central corneal thickness. Hum Genet 131**:** 1783-93.

Hysi, PG, Cheng, CY, Springelkamp, H, Macgregor, S, Bailey, JN, Wojciechowski, R, Vitart, V, Nag, A, Hewitt, AW, Hohn, R, Venturini, C, Mirshahi, A et al. 2014. Genome-wide analysis of multi-ancestry cohorts identifies new loci influencing intraocular pressure and susceptibility to glaucoma. Nat Genet 46**:** 1126-30.

Khor, CC, Ramdas, WD, Vithana, EN, Cornes, BK, Sim, X, Tay, WT, Saw, SM, Zheng, Y, Lavanya, R, Wu, R, Wang, JJ, Mitchell, P et al. 2011. Genome-wide association studies in Asians confirm the involvement of ATOH7 and TGFBR3, and further identify CARD10 as a novel locus influencing optic disc area. Hum Mol Genet 20**:** 1864-72.

Li, Z, Allingham, RR, Nakano, M, Jia, L, Chen, Y, Ikeda, Y, Mani, B, Chen, LJ, Kee, C, Garway-Heath, DF, Sripriya, S, Fuse, N et al. 2015. A common variant near TGFBR3 is associated with primary open angle glaucoma. Hum Mol Genet.

Lu, Y, Vitart, V, Burdon, KP, Khor, CC, Bykhovskaya, Y, Mirshahi, A, Hewitt, AW, Koehn, D, Hysi, PG, Ramdas, WD, Zeller, T, Vithana, EN et al. 2013. Genome-wide association analyses identify multiple loci associated with central corneal thickness and keratoconus. Nat Genet 45**:** 155-63.

Macgregor, S, Hewitt, AW, Hysi, PG, Ruddle, JB, Medland, SE, Henders, AK, Gordon, SD, Andrew, T, McEvoy, B, Sanfilippo, PG, Carbonaro, F, Tah, V et al. 2010. Genome-wide association identifies ATOH7 as a major gene determining human optic disc size. Hum Mol Genet 19**:** 2716-24.

Meguro, A, Inoko, H, Ota, M, Mizuki, N , Bahram, S 2010. Genome-wide association study of normal tension glaucoma: common variants in SRBD1 and ELOVL5 contribute to disease susceptibility. Ophthalmology 117**:** 1331-8.e5.

Nag, A, Venturini, C, Small, KS, Young, TL, Viswanathan, AC, Mackey, DA, Hysi, PG , Hammond, C 2014. A genome-wide association study of intra-ocular pressure suggests a novel association in the gene FAM125B in the TwinsUK cohort. Hum Mol Genet 23**:** 3343-8.

Nakano, M, Ikeda, Y, Tokuda, Y, Fuwa, M, Omi, N, Ueno, M, Imai, K, Adachi, H, Kageyama, M, Mori, K, Kinoshita, S , Tashiro, K 2012. Common variants in CDKN2B-AS1 associated with optic-nerve vulnerability of glaucoma identified by genome-wide association studies in Japanese. PLoS One 7**:** e33389.

Osman, W, Low, SK, Takahashi, A, Kubo, M , Nakamura, Y 2012. A genome-wide association study in the Japanese population confirms 9p21 and 14q23 as susceptibility loci for primary open angle glaucoma. Hum Mol Genet 21**:** 2836-42.

Ozel, AB, Moroi, SE, Reed, DM, Nika, M, Schmidt, CM, Akbari, S, Scott, K, Rozsa, F, Pawar, H, Musch, DC, Lichter, PR, Gaasterland, D et al. 2014. Genome-wide association study and meta-analysis of intraocular pressure. Hum Genet 133**:** 41-57.

Ramdas, WD, van Koolwijk, LM, Ikram, MK, Jansonius, NM, de Jong, PT, Bergen, AA, Isaacs, A, Amin, N, Aulchenko, YS, Wolfs, RC, Hofman, A, Rivadeneira, F et al. 2010. A genome-wide association study of optic disc parameters. PLoS Genet 6**:** e1000978.

Ramdas, WD, van Koolwijk, LM, Lemij, HG, Pasutto, F, Cree, AJ, Thorleifsson, G, Janssen, SF, Jacoline, TB, Amin, N, Rivadeneira, F, Wolfs, RC, Walters, GB et al. 2011. Common genetic variants associated with open-angle glaucoma. Hum Mol Genet 20**:** 2464-71.

Springelkamp, H, Hohn, R, Mishra, A, Hysi, PG, Khor, CC, Loomis, SJ, Bailey, JN, Gibson, J, Thorleifsson, G, Janssen, SF, Luo, X, Ramdas, WD et al. 2014. Meta-analysis of genome-wide association studies identifies novel loci that influence cupping and the glaucomatous process. Nat Commun 5**:** 4883.

Springelkamp, H, Mishra, A, Hysi, PG, Gharahkhani, P, Hohn, R, Khor, CC, Cooke Bailey, JN, Luo, X, Ramdas, WD, Vithana, E, Koh, V, Yazar, S et al. 2015. Meta-analysis of Genome-Wide Association Studies Identifies Novel Loci Associated With Optic Disc Morphology. Genet Epidemiol 39**:** 207-16.

Strange, A, Bellenguez, C, Sim, X, Luben, R, Hysi, PG, Ramdas, WD, van Koolwijk, LM, Freeman, C, Pirinen, M, Su, Z, Band, G, Pearson, R et al. 2013. Genome-wide association study of intraocular pressure identifies the GLCCI1/ICA1 region as a glaucoma susceptibility locus. Hum Mol Genet 22**:** 4653-60.

Takamoto, M, Kaburaki, T, Mabuchi, A, Araie, M, Amano, S, Aihara, M, Tomidokoro, A, Iwase, A, Mabuchi, F, Kashiwagi, K, Shirato, S, Yasuda, N et al. 2012. Common variants on chromosome 9p21 are associated with normal tension glaucoma. PLoS One 7**:** e40107.

Thorleifsson, G, Walters, GB, Hewitt, AW, Masson, G, Helgason, A, DeWan, A, Sigurdsson, A, Jonasdottir, A, Gudjonsson, SA, Magnusson, KP, Stefansson, H, Lam, DS et al. 2010. Common variants near CAV1 and CAV2 are associated with primary open-angle glaucoma. Nat Genet 42**:** 906-9.

Ulmer, M, Li, J, Yaspan, BL, Ozel, AB, Richards, JE, Moroi, SE, Hawthorne, F, Budenz, DL, Friedman, DS, Gaasterland, D, Haines, J, Kang, JH et al. 2012. Genome-wide analysis of central corneal thickness in primary open-angle glaucoma cases in the NEIGHBOR and GLAUGEN consortia. Invest Ophthalmol Vis Sci 53**:** 4468-74.

van Koolwijk, LM, Ramdas, WD, Ikram, MK, Jansonius, NM, Pasutto, F, Hysi, PG, Macgregor, S, Janssen, SF, Hewitt, AW, Viswanathan, AC, ten Brink, JB, Hosseini, SM et al. 2012. Common genetic determinants of intraocular pressure and primary open-angle glaucoma. PLoS Genet 8**:** e1002611.

Vitart, V, Bencic, G, Hayward, C, Skunca Herman, J, Huffman, J, Campbell, S, Bucan, K, Navarro, P, Gunjaca, G, Marin, J, Zgaga, L, Kolcic, I et al. 2010. New loci associated with central cornea thickness include COL5A1, AKAP13 and AVGR8. Hum Mol Genet 19**:** 4304-11.

Vithana, EN, Aung, T, Khor, CC, Cornes, BK, Tay, WT, Sim, X, Lavanya, R, Wu, R, Zheng, Y, Hibberd, ML, Chia, KS, Seielstad, M et al. 2011. Collagen-related genes influence the glaucoma risk factor, central corneal thickness. Hum Mol Genet 20**:** 649-58.

Wiggs, JL, Kang, JH, Yaspan, BL, Mirel, DB, Laurie, C, Crenshaw, A, Brodeur, W, Gogarten, S, Olson, LM, Abdrabou, W, DelBono, E, Loomis, S et al. 2011. Common variants near CAV1 and CAV2 are associated with primary open-angle glaucoma in Caucasians from the USA. Hum Mol Genet 20**:** 4707-13.

Wiggs, JL, Yaspan, BL, Hauser, MA, Kang, JH, Allingham, RR, Olson, LM, Abdrabou, W, Fan, BJ, Wang, DY, Brodeur, W, Budenz, DL, Caprioli, J et al. 2012. Common variants at 9p21 and 8q22 are associated with increased susceptibility to optic nerve degeneration in glaucoma. PLoS Genet 8**:** e1002654.
